# Jun dimerization protein 2 is a critical component of the Nrf2/MafK complex regulating the response to ROS homeostasis

**DOI:** 10.1038/cddis.2013.448

**Published:** 2013-11-14

**Authors:** S Tanigawa, C H Lee, C S Lin, C C Ku, H Hasegawa, S Qin, A Kawahara, Y Korenori, K Miyamori, M Noguchi, L H Lee, Y C Lin, C L Steve Lin, Y Nakamura, C Jin, N Yamaguchi, R Eckner, D-X Hou, K K Yokoyama

**Affiliations:** 1 Faculty of Agriculture, Kagoshima University, Kagoshima, Japan; 2 Department of Dermatology, Kaohsiung Chang Gung Memorial Hospital and Chang Gung University, College of Medicine, Kaohsiung, Taiwan; 3 Graduate Institute of Medicine, Kaohsiung Medical University, Kaohsiung, Taiwan; 4 Graduate School of Pharmaceutical Sciences, Chiba University, Chiba, Japan; 5 Hunan Agriculture University, Changsha, China; 6 RIKEN BioResource Center, Tsukuba, Japan; 7 School of Dentistry, Kaohsiung Medical University, Kaohsiung, Taiwan; 8 Cancer Center, Kaohsiung Medical University Hospital, Ksohsiung, Taiwan; 9 Department of Environmental Medicine, NYU School of Medicine, Tuxedo, NY, USA; 10 Department of Biochemistry and Molecular Biology, Rutgers New Jersey Medical School, Rutgers, The State University of New Jersey, Newark, NJ, USA

**Keywords:** JDP2, Nrf2–MafK, ROS regulation, antioxidant enzymes, antioxidation

## Abstract

Oxidative stress and reactive oxygen species (ROS) are associated with diseases such as cancer, cardiovascular complications, inflammation and neurodegeneration. Cellular defense systems must work constantly to control ROS levels and to prevent their accumulation. We report here that the Jun dimerization protein 2 (JDP2) has a critical role as a cofactor for transcription factors nuclear factor-erythroid 2-related factor 2 (Nrf2) and small Maf protein family K (MafK) in the regulation of the antioxidant-responsive element (ARE) and production of ROS. Chromatin immunoprecipitation–quantitative PCR (qPCR), electrophoresis mobility shift and ARE-driven reporter assays were carried out to examine the role of JDP2 in ROS production. JDP2 bound directly to the ARE core sequence, associated with Nrf2 and MafK (Nrf2–MafK) via basic leucine zipper domains, and increased DNA-binding activity of the Nrf2–MafK complex to the ARE and the transcription of ARE-dependent genes. In mouse embryonic fibroblasts from *Jdp2*-knockout (*Jdp2* KO) mice, the coordinate transcriptional activation of several ARE-containing genes and the ability of Nrf2 to activate expression of target genes were impaired. Moreover, intracellular accumulation of ROS and increased thickness of the epidermis were detected in *Jdp2* KO mice in response to oxidative stress-inducing reagents. These data suggest that JDP2 is required to protect against intracellular oxidation, ROS activation and DNA oxidation. qPCR demonstrated that several Nrf2 target genes such as heme oxygenase-1, glutamate–cysteine ligase catalytic and modifier subunits, the notch receptor ligand jagged 1 and NAD(P)H dehydrogenase quinone 1 are also dependent on JDP2 for full expression. Taken together, these results suggest that JDP2 is an integral component of the Nrf2–MafK complex and that it modulates antioxidant and detoxification programs by acting via the ARE.

A range of factors, including xenobiotics, drugs, heavy metals and ionizing radiation, can induce oxidative stress, which leads to the generation of reactive oxygen species (ROS) and electrophiles.^1, 2, 3, 4^ ROS levels are strictly controlled by an antioxidant program that responds to cellular stressors. The coordinate induction of the genes encoding the Phase II detoxification and antioxidant enzymes is governed by the core sequence (5′-G/ATGACNNNGC-3′) located in the gene regulatory regions and is termed antioxidant-responsive element (ARE)–electrophile responsive elements.^2, 5, 6, 7, 8, 9, 10^ The key factor involved in the binding to AREs is the nuclear factor-erythroid 2-related factor 2 (Nrf2). Nrf2 forms heterodimeric complexes with a small Maf protein (MafG, MafK or MafF) for high-affinity binding to AREs.^6, 10, 11, 12, 13^ The Nrf2–ARE axis is absolutely essential for the induction of genes encoding antioxidant and detoxification enzymes, and it contributes to the regulation of cell growth, differentiation and survival after oxidative stress.

The c-Jun dimerization protein 2 (JDP2) is a basic zipper region (bZIP) repressor protein that is highly expressed in the brain and lung.^14, 15, 16^ JDP2 can homodimerize and can form heterodimers with other AP-1 family members through leucine zipper motif.^14, 15, 16, 17, 18^ A basic domain is responsible for its direct association with AP-1 and CRE sites of DNA.^14, 15^ The mechanisms by which JDP2 represses AP-1 transcription may involve competition for DNA binding,^14^ inactive heterodimer formation,^14^ indirect recruitment of histone deacetylase 3,^19^ nucleosome assembly activity, inhibition of histone acetylation^20^ and potential competition with JNK phosphorylation.^21^ Knockout (KO) of the *Jdp2* gene affects adipocyte and neutrophil differentiation,^22, 23^ resistance to replicative senescence,^24^ cell cycle arrest and regulation of cyclin A2 and p53.^16, 24, 25^ As JDP2 is also a member of the bZIP family of transcription factors, we examined whether JDP2 binds to Maf-family and/or Nrf2 proteins, and whether it can regulate ARE-dependent genes encoding antioxidant and detoxification enzymes.

Here we report that JDP2 associates with the ARE and acts as a newly identified cofactor of the Nrf2–MafK complex to modulate ARE-mediated gene expression and ROS production. Our results provide evidence that JDP2 has a critical role in the cellular adaptive response to ROS and electrophiles generated by cellular stimuli.

## Results

### ROS production and antioxidant activity

**Figure 1:**
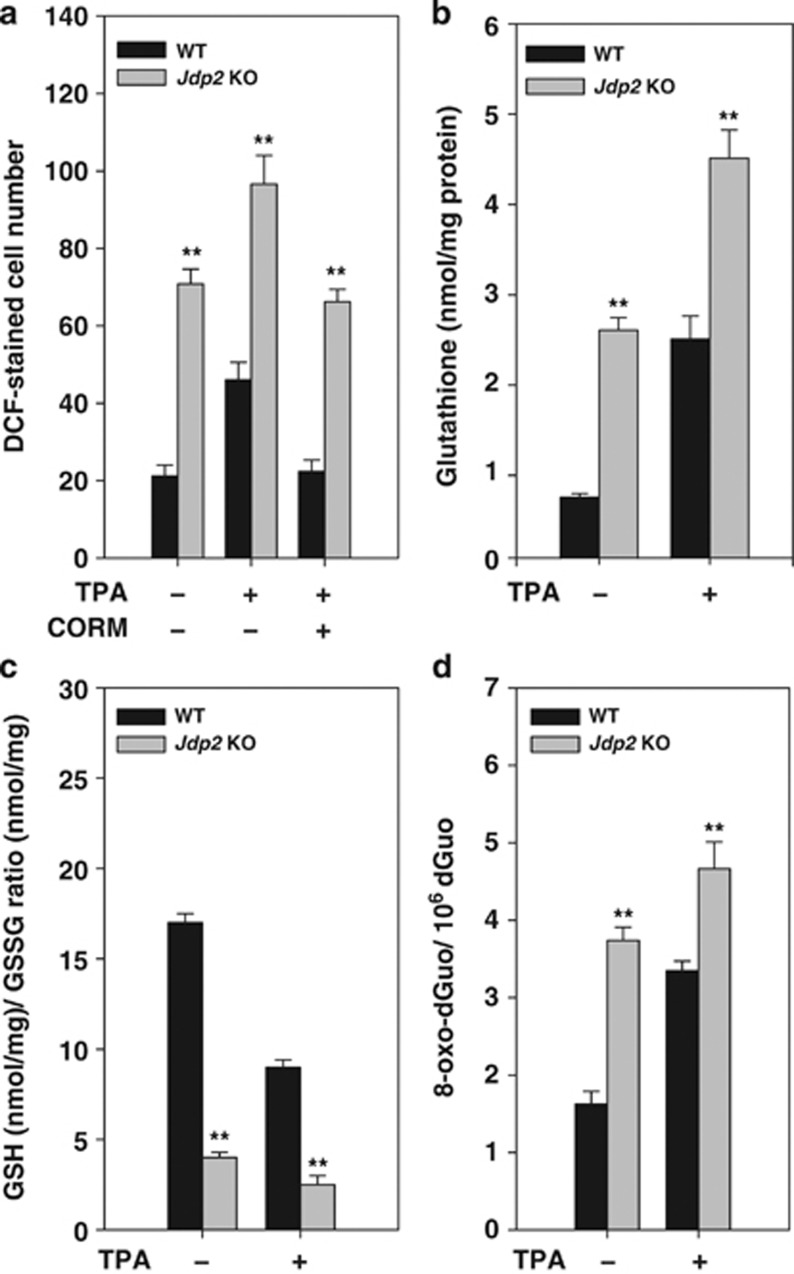
JDP2 controls ROS production. (**a**) MEFs were seeded (5 × 10^3^ per well) into eight-chambered cover glasses immersed in 500 *μ*l of 10% FBS–DMEM and exposed or unexposed to TPA (10^−6^ M) or with or without 50 nmol/l of CORM. Intracellular ROS levels were measured using H_2_DCFDA staining, as described in the Materials and Methods. (**b**) Determination of the concentration of total glutathione in WT and *Jdp2* KO MEFs incubated with TPA (10^−6 ^M) for 24 h. (**c**) Determination of the GSH/GSSG ratio in WT and *Jdp2* KO MEFs incubated with TPA (10^–6^ M) for 24 h. The ration of GSH/GSSG was calculated in the Materials and Methods. (**d**) 8-Oxo-dGuo levels in WT and *Jdp2* KO MEFs in the presence or absence of TPA (10^–6^ M) for 24 h. Data are presented as mean±S.D. (*n*=3). The data were analyzed using Student's *t*-test; **P<0.01

As JDP2 is a member of the stress-induced AP-1 protein family,^15^ we examined the role of JDP2 in ROS production and the antioxidant response. Strikingly, the basal ROS level was about 3.4-fold higher in *Jdp2* KO mouse embryonic fibroblasts (MEFs) compared with wild-type (WT) MEFs. 12-*O*-tetradecanoylphorbol-13-acetate (TPA) treatment further increased ROS levels in *Jdp2* KO and WT MEFs (Figure 1a). As expected, exposure of MEFs to the ROS inhibitor CO-releasing molecule tricarbonyldichlororuthenium (CORM) abolished the TPA-induced increase in ROS production. We conclude that deletion of JDP2 leads to higher level of ROS in both basal and TPA-induced states, and thus JDP2 function is required to keep ROS levels low.

As ROS alterations can affect the intracellular redox state,^26^ we next measured the ratio of reduced to oxidized glutathione (GSH/GSSG) in WT and *Jdp2* KO MEFs.^27^ Although the level of total glutathione was 3.8-fold higher in *Jdp2* KO MEFs compared with WT MEFs (Figure 1b), the GSH/GSSG ratio was reduced to 23.8% of that seen in WT MEF cells, thus also indicating a more oxidized intracellular environment in MEFs lacking *Jdp2* (Figure 1c). Antioxidant reagents such as sulforaphane (SFN)^28, 29^ and tertiary butylhydroquinone (tBHQ)^30^ increased the ratio of GSH/GSSG to higher levels in *Jdp2* KO MEFs than WT MEFs (Supplementary Figure S1). The level of 8-oxo-7,8-dihydro-2′-deoxyguanosine (8-oxo-dGuo), one of the major products of DNA oxidation, was 2.4-fold higher in *Jdp2* KO MEFs compared with WT MEFs, and TPA induced moderate production of 8-oxo-dGuo in both WT and *Jdp2* KO MEFs (Figure 1d). Thus, *Jdp2*-deficient MEFs exhibited higher levels of ROS, 8-oxo-dGuo and total glutathione, which was more oxidized than that in WT MEFs. All of these results strongly suggest that JDP2 function is required to hold ROS levels in check.

### TPA-mediated ARE transactivation

**Figure 2:**
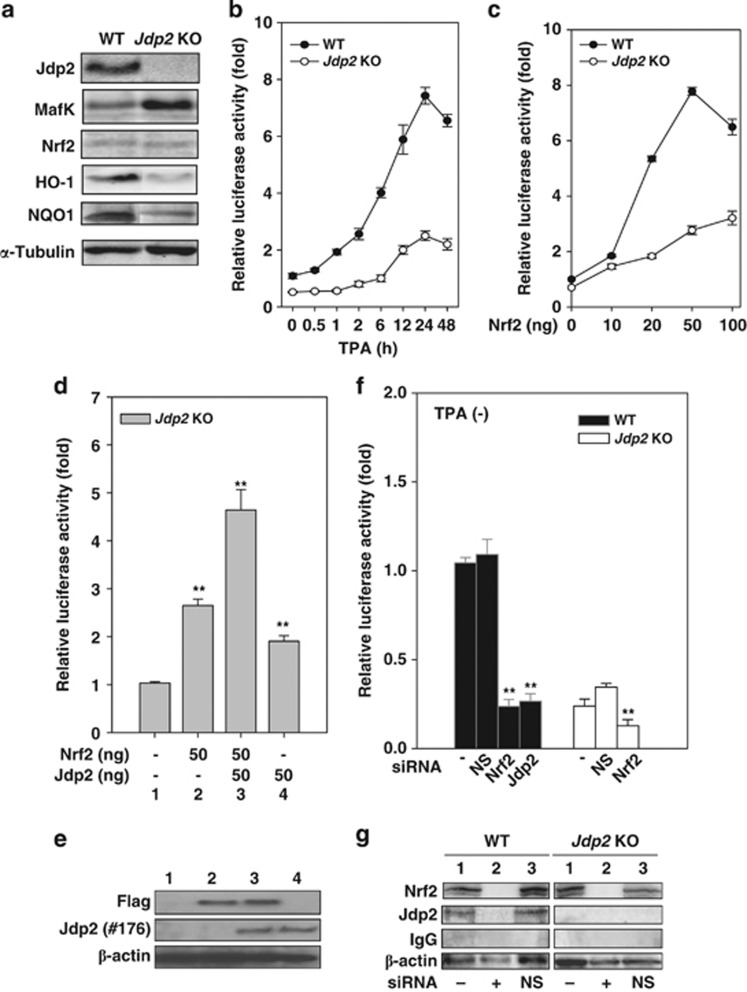
JDP2 is required for ARE activation. (**a**) Protein detection in WT and *Jdp2* KO MEFs using western blotting. The cellular lysates from WT and *Jdp2* KO MEFs (40 *μ*g) were separated on SDS-PAGE and then transferred onto a membrane. Jdp2, MafK, Nrf2, HO-1, NQO1 and *α*-tubulin were immunodetected using specific antibodies. (**b**) Relative NQO1 promoter activity in WT and *Jdp2* KO MEFs in the presence of TPA at the indicated exposure times. After 24 h of culture, TPA (10^–6^ M) was added, transfectants with pGL4–hQR25–luciferase were incubated for an additional 48 h and luciferase activity was measured (*n*=3) as described in Materials and Methods. (**c**) Effect of Nrf2 on NQO1 promoter activity. pGL4–hQR25–luciferase (400 ng) plus 0–100 ng of pcDNA3–Nrf2 were transfected into WT and *Jdp2* KO MEFs (5 × 10^4^). One day after transfection, cells were collected and luciferase activity was measured. Values from a representative experiment are given as mean±S.D. (*n*=3). (**d**) Effect of JDP2 on ARE activity in the presence of Nrf2. *Jdp2* KO MEFs (5 × 10^4^) was transfected with 400 ng of pGL4–hQR25–luciferase, 50 ng of FLAG_S–Nrf2 and pcDNA–Jdp2, respectively, as indicated. One day after transfection, cells were collected and luciferase activity was measured (*n*=3). **P<0.01. (**e**) Protein detection of FLAG–Nrf2 and JDP2 in WT and *Jdp2* KO MEFs using western blotting. The cellular lysates from WT and *Jdp2* KO MEFs (40 *μ*g) were separated on SDS-PAGE and then transferred onto a membrane. FLAG, Jdp2 and *β*-actin were immunodetected using specific antibodies, which were described in Materials and Methods. Lane number corresponded to each lane in **d**. (**f**) Effect of siRNA specific for Nrf2 and Jdp2 on ARE activity. WT and *Jdp2* KO MEFs (5 × 10^4^ cells) were transfected with 30 pmol of siRNA specific for Nrf2 or Jdp2 and 200 ng of pGL4–hQR25–luciferase plasmid as described in the text. After exposure for 30 h, luciferase activity was measured (*n*=3). The same amount of nonspecific double-stranded RNA was used as a negative control (NS). **P<0.01. (**g**) Inhibition of the expression of Nrf2 and Jdp2 proteins by siRNA. WT and *Jdp2* KO MEFs (5 × 10^4^) were transfected with 30 pmol of active siRNA and the control siRNA, together with reporter plasmids. After treatment for 30 h, cell extracts were analyzed by immunoblotting using Nrf2, Jdp2, IgG or *β*-actin antibodies. Lane 1, without siRNA; lane 2, with siRNA; and lane 3, with NS siRNA

An immunoblot survey of several of the key components involved in controlling ROS level revealed that the basal levels of the hemoxigenase-1 (HO-1) protein and NAD(P)H dehydrogenase quinone 1 (NQO1) were reduced more than five fold in *Jdp2* KO MEFs compared with WT MEFs respectively (Figure 2a). The Nrf2 expression level was almost the same, whereas MafK expression levels were 3.5-fold higher in *Jdp2* KO MEFs compared with WT MEFs (Figure 2a). We conclude that lower HO-1 and NQO1 protein levels may, in part, explain the higher ROS levels in *Jdp2* KO MEFs.

In an attempt to examine the role of JDP2 in antioxidant response, a luciferase reporter containing ARE derived from NQO1 promoter was transfected in WT and *Jdp2* KO MEFs. As expected, the ARE activity in *Jdp2* KO MEFs was decreased >50% relative to that of WT MEFs. TPA stimulated ARE–luciferase activity by 7.2-fold in WT MEFs after 24 h and only in the order of 4.2-fold in *Jdp2* KO MEFs (Figure 2b). The ROS inhibitor CORM abolished the TPA-induced ARE activity (Supplementary Figure S2a). We conclude that JDP2 is required for antioxidant activity in the basal and induced state.

As Nrf2 acts as the key transcription factor in triggering ROS-induced gene expression, we investigated Nrf2 activity in *Jdp2* KO MEFs. An ARE-driven luciferase reporter plasmid was transfected into WT and *Jdp2* KO MEFs together with increasing amounts of Nrf2. Nrf2 increased ARE promoter activity in WT MEFs by 7.8-fold and ∼3-fold in *Jdp2* KO MEFs (Figure 2c). We next tried to rescue Nrf2-mediated transactivation in *Jdp2* KO MEFs by cotransfecting a JDP2 expression plasmid together with Nrf2 at the optimal dose (50 ng). We found that JDP2 increased the Nrf2-mediated ARE promoter activity (Figures 2d and e). By contrast, MafK inhibited Nrf2-dependent ARE activity in a dose-dependent manner (Supplementary Figure S2b). Similarly, the ARE–luciferase activity was increased by the introduction of expression vectors of *Nrf2* or *Jdp2* in WT MEFs and *Jdp2* KO MEFs; however, the MafK repressed the ARE–luciferase activity (Supplementary Figures S2c and d).

To complement these experiments, we used small interfering RNAs (siRNAs) to knock down (KD) the expression of Nrf2 and Jdp2 in WT MEFs. ARE reporter gene activity was decreased by approximately four fold after introduction of Nrf2 KD or Jdp2 KD in WT MEFs in the absence (Figure 2f) and presence of TPA (Supplementary Figure S3), respectively. Similarly, the ARE–luciferase activity was decreased after Nrf2 KD in *Jdp2* KO MEFs and was unaffected by nonspecific siRNA (NS siRNA). Each siRNA against Nrf2 or Jdp2 reduced the expression of the respective endogenous protein (Figure 2g). These results are consistent with the view that JDP2 function is critical for basal and for induced Nrf2 transcriptional activity.

### SFN-induced ARE–luciferase activity and epidermal thickness

**Figure 3:**
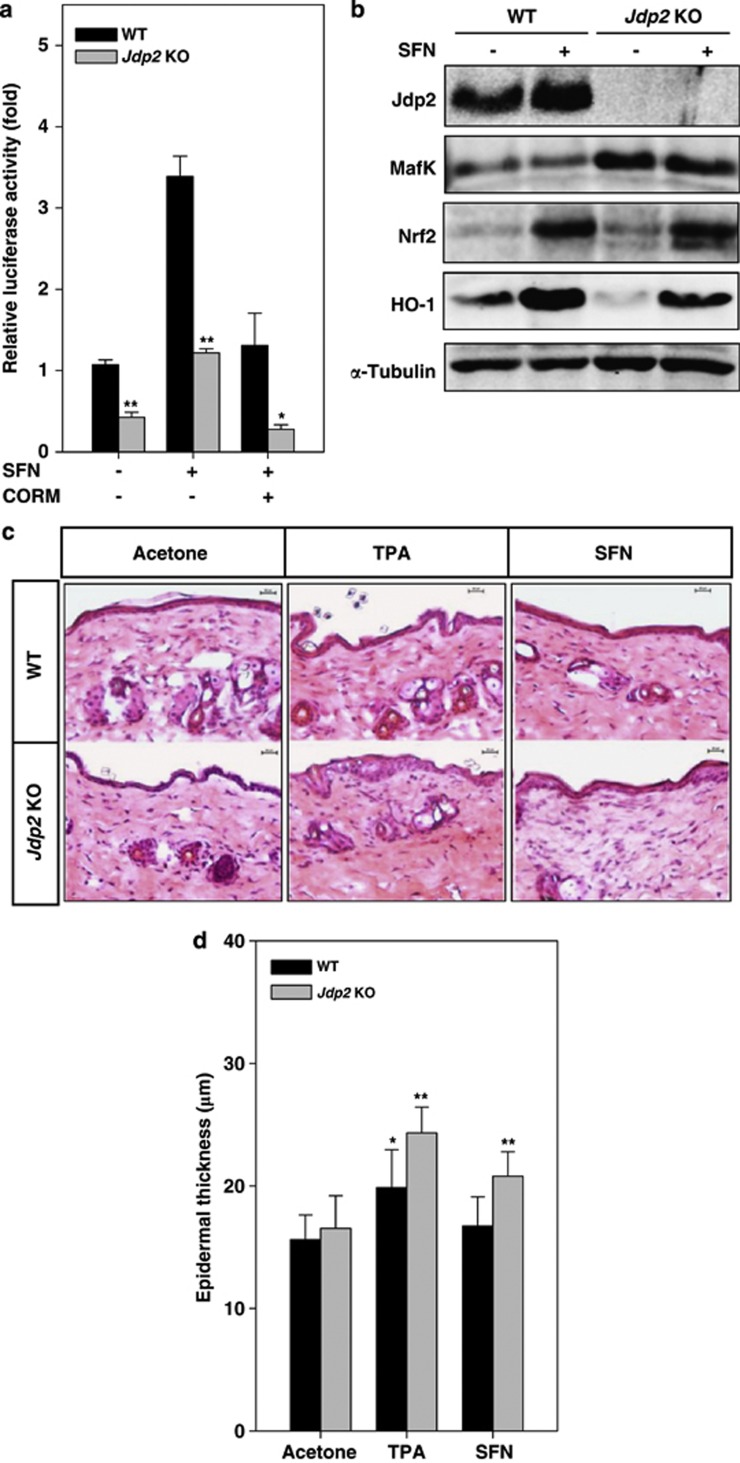
JDP2 is involved in SFN-induced ARE transactivation. (**a**) ARE-driven transcriptional activity in WT and *Jdp2* KO MEFs after treatment with or without SFN (5 × 10^–6^ M) in the presence or absence of 50 nmol/l of CORM. The pGL4–hQR25–luciferase plasmid was transfected into WT and *Jdp2* KO MEFs. After 24 h of culture, SFN (5 *μ*M) was added and cells were cultured for an additional 12 h. Cellular luciferase activity was measured (*n*=3) as described in Materials and Methods. *P<0.05; **P<0.01. (**b**) Protein detection in WT and *Jdp2* KO MEFs treated with SFN. After 24 h of culture, SFN (10 *μ*M) was added and cells were cultured for an additional 12 h. Cellular lysates from WT and *Jdp2* KO MEFs (40 *μ*g) were separated on SDS-PAGE and transferred onto membranes, and the antibody specific for each protein of interest was used for immunodetection. (**c**) Comparison of epidermal thickening induced by TPA and SFN in WT and *Jdp2* KO mice. WT and *Jdp2* KO mice were painted with acetone or TPA (8.1 nmol in 100 *μ*l of acetone) or SFN (15 nmol in 100 *μ*l of acetone) twice, and skin tissue sections were prepared and stained with hematoxylin and eosin. Each group included five mice. The epidermal thickness of the skin was measured at 10 sites of an area randomly selected and was defined as the distance from the basal layer to the corneal layer. The results for epidermal thickening are quantified in (**d**) (*n*=3). *P<0.05; **P<0.01

To determine whether JDP2 is involved in SFN-induced ARE transactivation, we transfected an ARE–luciferase plasmid into both WT and *Jdp2* KO MEFs, and treated the cells for 12 h with SNF. SNF increased ARE–luciferase transcription by 3.4-fold in WT MEFs. In *Jdp2* KO MEFs, however, basal levels were again lower (39.5% of WT levels) and SFN increased ARE transcription by ∼3-fold (Figure 3a). The expression of the Nrf2 protein in WT and *Jdp2* KO MEFs was increased by 4.0- to 4.5-fold in response to SFN, but the relative level of the MafK protein was not upregulated by SFN in WT or *Jdp2* KO MEFs (Figure 3b). However, in agreement with the data shown in Figure 2a, the level of MafK was 2.5-fold higher in *Jdp2* KO MEFs than in WT MEFs.

We next examined the effect of SFN on epidermal thickening (Figure 3c). Analysis of the skin of *Jdp2* KO mice provided evidence that the *in vivo r*esponse to TPA and SFN is also altered by the loss of JDP2. Comparison of the skin of TPA-treated WT and *Jdp2* KO mice revealed that the epidermal thickness of the hairless skin from *Jdp2* KO mice was increased by about 1.45-fold after TPA treatment (Figure 3d).^16^ Administration of SFN to WT mice had only a limited effect, whereas administration of SFN to *Jdp2* KO mice induced a significant epidermal thickening (1.2-fold; Figure 3d). These data indicated that JDP2 antagonizes TPA-dependent *in vivo* cell proliferation and its related activity in the epidermis thickening.

### The zipper region of JDP2 is required for *in vitro* association with ARE

**Figure 4:**
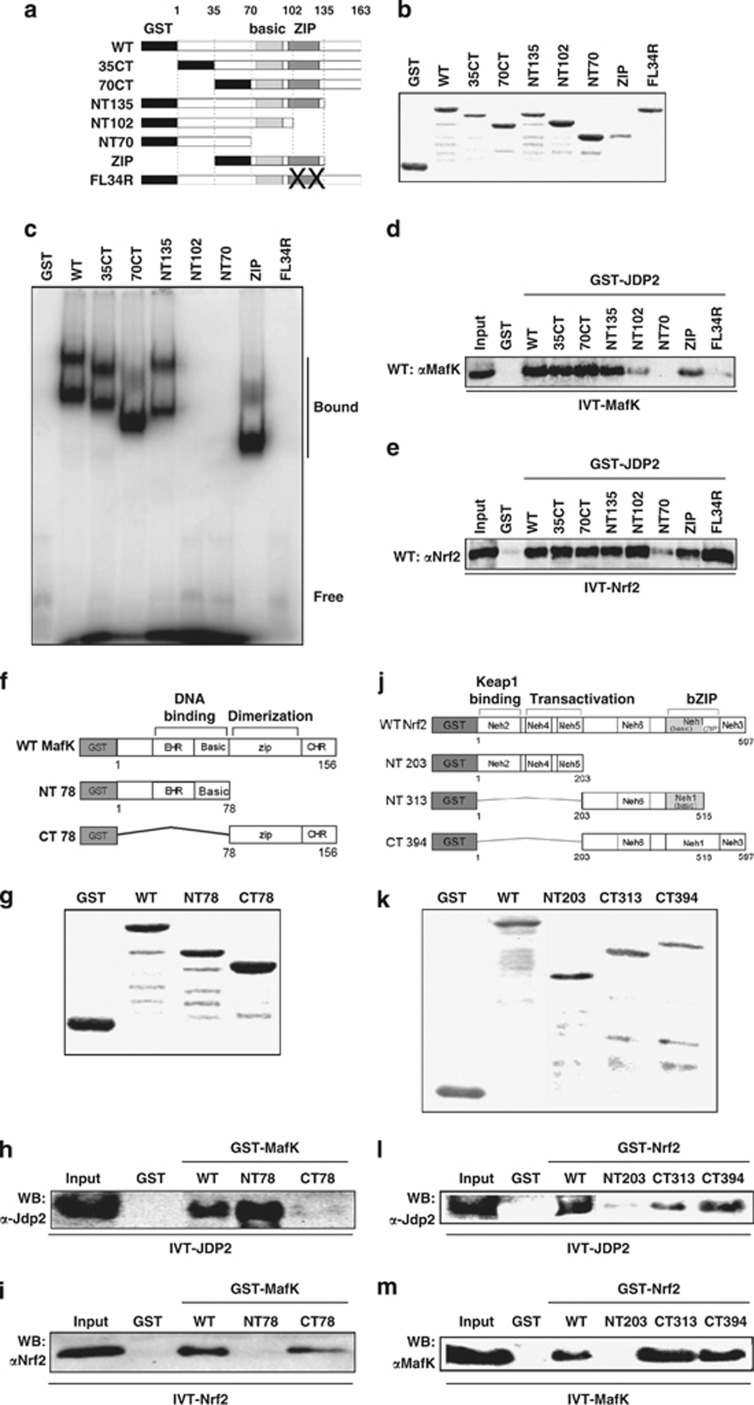
Reciprocal interaction of JDP2, Nrf2 and MafK *in vitro*. (**a**) The structure of GST–JDP2 proteins used for EMSA assay *in vitro*. The structures of various mutant forms of JDP2 are shown below. (**b**) Expression of the GST–JDP2 proteins. The GST–JDP2 proteins were detected on 10% SDS-PAGE after purification with GST affinity resins. (**c**) EMSA assay of the JDP2 deletion mutants with human NQO1–ARE. EMSA assay was performed using a series of deletion mutants of the Jdp2 protein, as indicated in Materials and Methods. The upper bands of the DNA–protein complexes seem to be dimer forms. (**d**) The bZIP domain of JDP2 is recruited and binds MafK. The full-length rat MafK (*in vitro-*translated MafK; IVT–MafK) was expressed using an *in vitro* transcription–translation system without [^35^S]-methionine as recommended in the manufacturer's protocol. Affinity-resin-purified GST or GST–JDP2 fusion variant proteins were mixed with IVT–MafK. The bound MafK was applied to SDS-PAGE and immunodetected using a MafK-specific antibody. (**e**) The basic domain of JDP2 is recruited and binds Nrf2. The IVT–Nrf2 protein was incubated with GST–JDP2 and a protein-binding assay was performed as described in **c**. (**f**) The structure of mutant forms of various GST–MafK proteins used for EMSA assay *in vitro*. (**g**) Expression of the GST–MafK proteins. The GST–MafK was detected on 10% SDS-PAGE after purification with GST affinity resins. (**h**) The DNA-binding domain of MafK is recruited and binds JDP2. The full-length mouse JDP2 (IVT–JDP2) was expressed, using an *in vitro* transcription–translation system without [^35^S]-methionine, as recommended in the manufacturer's protocol. Affinity-resin-purified GST or GST–MafK fusion variant proteins were mixed with IVT–JDP2. The bound JDP2 was applied to SDS-PAGE and immunodetected using a JDP2-specific antibody. (**i**) The ZIP domain of MafK is recruited and binds Nrf2. The structure and expression of GST–MafK and IVT–Nrf2 were described above. The bound Nrf2 was immunodetected using an Nrf2-specific antibody. (**j**) The structure of various mutant forms of the GST–Nrf2 protein used for EMSA assay *in vitro*. (**k**) Expression of the GST–Nrf2 protein. The GST–Nrf2 proteins were detected on 10% SDS-PAGE after purification with GST affinity resins. (**l**) The C-terminal and half-bZIP domain of Nrf2 are recruited and bind JDP2. The full-length mouse JDP2 (IVT-Jdp2) was expressed using an *in vitro* transcription–translation system without [^35^S]-methionine. GST or GST–MafK fusion proteins were mixed with IVT–Jdp2. After washing, bound proteins were applied on SDS-PAGE and immunodetected using a Jdp2-specific antibody. (**m**) The bZIP and Neh 6 domains of Nrf2 are associated with MafK. The IVT–MafK protein and GST–Nrf2 proteins were incubated and the bound MafK was immunodetected using a MafK-specific antibody

To identify the domain of JDP2 that is involved in ARE binding, we used a series of deletion mutants of JDP2 and examined their binding to the ARE (Figure 4a). Glutathione *S*-transferase (GST)-fused JDP2 mutant proteins were prepared (Figure 4b) and then analyzed using an electrophoresis mobility shift assay (EMSA) with a [^32^P]-labeled ARE probe (Figure 4c). JDP2 mutant protein without basic and bZIP domains (NT70) did not bind to the ARE, neither did the mutant with a basic domain alone (NT102) nor the ZIP mutant (FL34R). These data indicate that the zipper domain of JDP2, in particular the leucine residues located at positions 114 and 121, is critical for binding to the ARE–DNA core.

### Cooperation of JDP2, MafK and Nrf2 toward ARE binding

To study the reciprocal interactions of JDP2, MafK and Nrf2 *in vitro*, we examined the activity of heterodimer formation using each recombinant GST–protein and protein synthesized by the *in vitro* transcription and translation (IVT–protein) system. The ZIP domain and the basic domain of JDP2 were required for the interaction with MafK and Nrf2 protein, respectively (Figures 4d and e). Thus, JDP2 uses distinct domains to interact with either MafK or Nrf2. We also prepared a series of deletion mutants of GST–MafK (Figures 4f and g) and GST–Nrf2 (Figures 4j and k), and examined their interaction. The DNA-binding domain and the C-terminal CHR region containing the zipper region of MafK were sufficient for interaction with JDP2 and Nrf2, respectively (Figures 4h and i), again suggesting that MafK uses distinct domains for binding to either JDP2 or Nrf2. Moreover, the C-terminal region containing the basic half region and the Neh6 region of Nrf2 were critical for the interaction with both JDP2 and MafK, respectively (Figures 4l and m). Accordingly, Nrf2 uses the same domain to bind JDP2 or MafK, and may therefore only be able to interact with one of the two proteins at a given time.

Both JDP2 and MafK bound to ARE–DNA, and both were able to interact with Nrf2 *in vitro*. An *in vitro* EMSA assay using each IVT–protein also showed that Nrf2 is not a DNA-binding protein but interacts with JDP2 (Supplementary Figure S4; lanes 1 and 4–6) or JDP2/MafK (lanes 10–15) to form the large complex with the higher DNA-binding activity.

### DNA-binding activity

**Figure 5:**
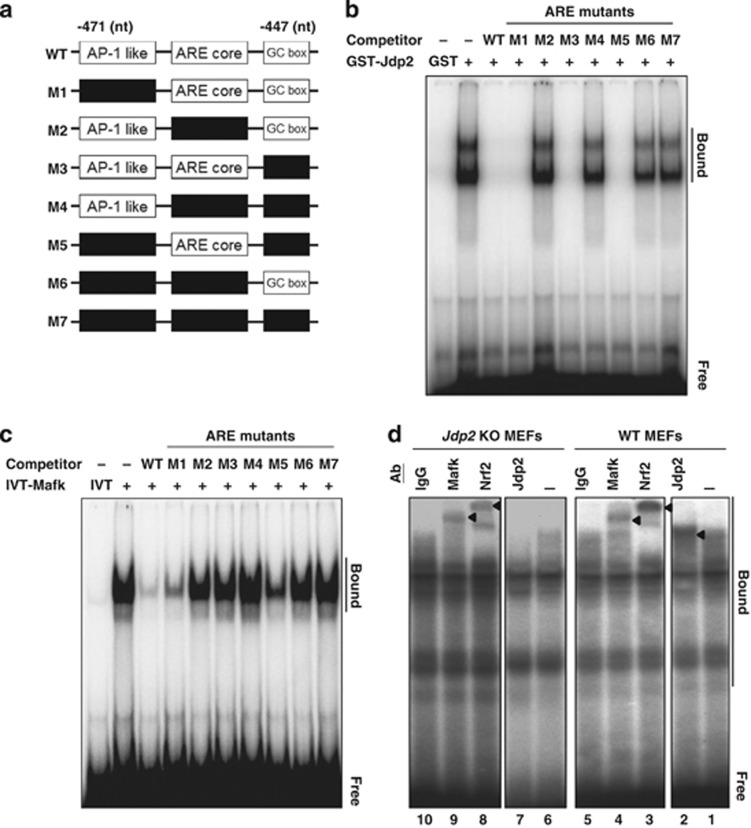
Cooperative binding of JDP2, MafK and Nrf2 to the ARE. (**a**) Tentative location of *cis*-elements in the ARE promoter region and its deletions mutants used for EMSA assay. AP-1-like (nt −471 to −466), ARE core (−462 to −456) and GC box (−455 to −453) elements are shown in white rectangular boxes. The deleted elements are shown in black rectangular boxes. The nucleotide sequences of the mutants (M1 to M7) were listed in the Supplementary Table I. (**b**) Competitive EMSA assay of JDP2 with ARE mutants. EMSA reactions were performed in the presence or absence of a competitive ARE as described in Materials and Methods, using [*γ*-^32^P]-labeled double-stranded ARE oligonucleotides. GST–JDP2 and GST proteins were purified using GST affinity resins. (**c**) Competitive EMSA assay of MafK with ARE mutants. IVT–MafK proteins were incubated with an ARE probe in the presence or absence of competitive ARE mutants and EMSA assay was performed as described in Materials and Methods. IVT alone was used as a negative control. (**d**) Supershift EMSA assay with nuclear extracts (NEs) from WT MEFs and *Jdp2* KO MEFs using the DNA probe of human NQO1–ARE. NEs from WT MEFs and *Jdp2* KO MEFs were incubated without (lanes 1 and 6) and with antibodies specific for Jdp2 (lanes 2 and 7), Nrf2 (lanes 3 and 8), MafK (lanes 4 and 9) and IgG (lane 5 and 10). ‘Bound' indicates the supershifted DNA–protein complexes and ‘Free' indicates the ARE–DNA probe

A series of deletion mutants corresponding to the AP-1-like element, ARE core and GC box motifs were constructed derived from the human *NQO1* gene, and EMSA assay using recombinant JDP2 and MafK proteins was performed (Figure 5a). The cold competitors of M1, M3, M5 and WT blocked the binding of JDP2, whereas cold competitors of M2, M4, M6 and M7 did not compete with the binding of JDP2 (Figure 5b). Thus, based on the results of this competitive DNA-binding assay, JDP2 binds to the ARE core (5′-TGACTCAGC-3′) of the complete ARE (−471 to −447) element. Conversely, cold competitors of the ARE core plus GC box (M1) and WT inhibited the binding of MafK to the ARE, whereas those of the ARE core alone (M5) inhibited the binding of MafK to the ARE partially and cold competitors containing an AP-1-like element or GC box (M2, M3, M4, M6 and M7) did not compete with this binding (Figure 5c). These data indicate that MafK bound both the ARE core and the GC box elements but not the AP-1-like element.

We next examined the presence of Nrf2, MafK and JDP2 in the DNA–protein complex from WT MEFs and *Jdp2* KO MEFs using the WT ARE (Figure 5a top) as a probe in an EMSA assay (Figure 5d). In WT MEFs, a prominent supershift was detected when antibodies against JDP2 (lane 2), Nrf2 (lane 3) or MafK (lane 4), but not by control IgG (lane 5) were added. By contrast, in *Jdp2* KO MEFs, only antibodies against MafK and Nrf2 showed prominent supershifts, and antibodies against JDP2 did not show a supershift (lanes 7–9). These findings suggest that JDP2, MafK and Nrf2 are present in the DNA–protein complex formed by extracts prepared from WT MEFs. By contrast, extracts prepared from *Jdp2* KO MEFs do not show a supershift with Jdp2 antibodies as expected, but addition of Nrf2 and MafK antibodies induced supershifts.

We also used 293T cells, which had been transfected with expression plasmids coding for FLAG-tagged Nrf2, JDP2 and MafK. Nuclear extracts were prepared for each, and the immunoprecipitates with anti-FLAG were immunoblotted by the respective antibodies against Nrf2, MafK and JDP2 (Supplementary Figure S5). The results of these experiments confirm the ability of Nrf2 and MafK, Nrf2 and JDP2, and JDP2 and MafK to interact with each other.

### Colocalization and recruitment of Nrf2, MafK and JDP2 to the ARE

We first carried out immunolocalization studies using untransfected HeLa cells. Signals corresponding to Nrf2 were localized in the regions of decondensed chromatin and presented mainly as nuclear foci (Supplementary Figure S6a). By contrast, the signals for JDP2 and MafK were detected in most regions of nuclei (Supplementary Figure S6b). Most signals of Nrf2 and MafK colocalized with those of JDP2. Thus, purely based on their intranuclear location, a triple complex encompassing Nrf2, MafK and JDP2 has the potential to form in the nuclei of HeLa cells, or dimers such as MafK-Nrf2 and JDP2-Nrf2 also have a possible interaction.

**Figure 6:**
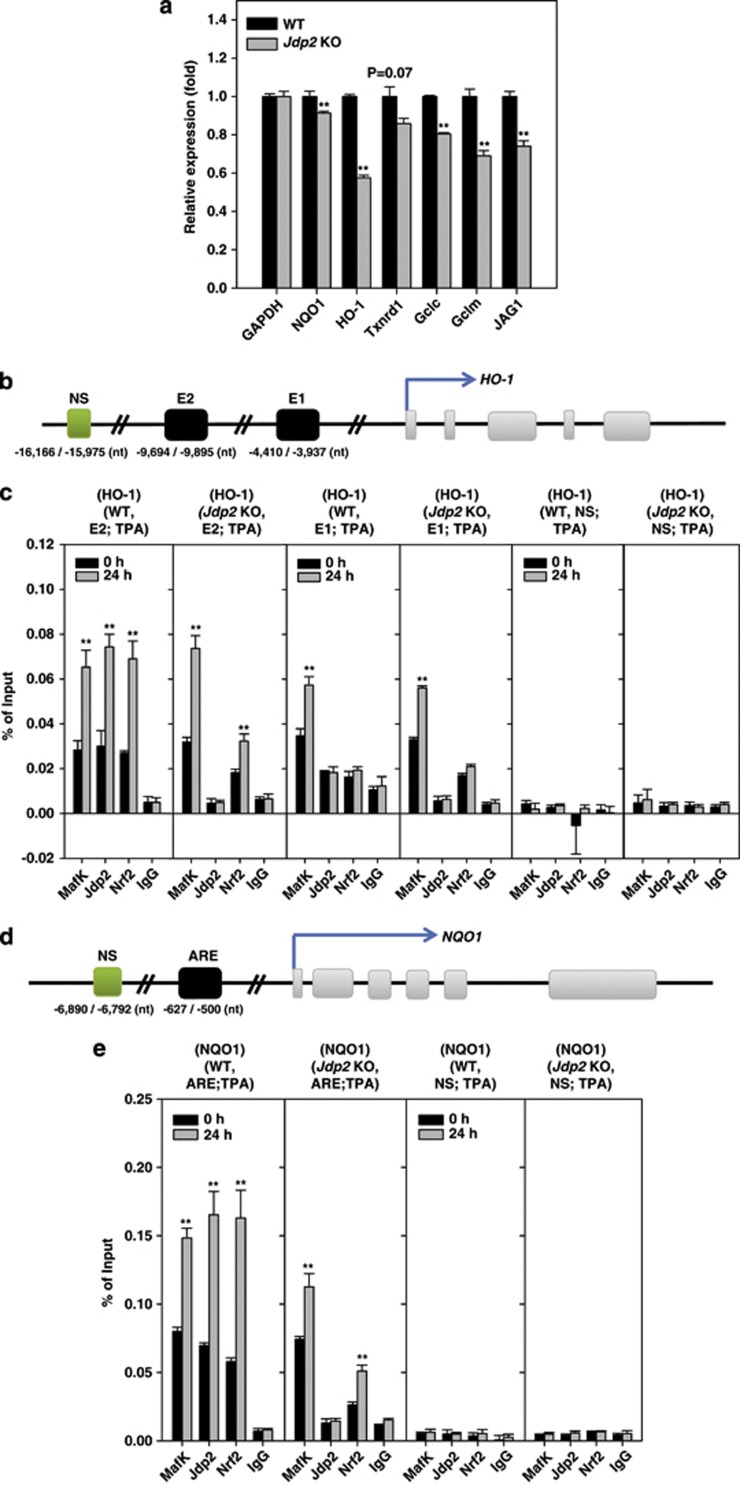
JDP2 regulates the expression of Nrf2 target genes. (**a**) qPCR analysis of Nrf2 target genes in WT and *Jdp2* KO MEFs. The expression levels determined are relative to the level of GAPDH. Error bars denote the S.D. from duplicate reactions by real-time PCR as described in Materials and Methods. The primer sequences of each Nrf2 target gene are listed in Supplementary Table SIII. *P<0.05; **P<0.01. (**b**–**e**) The ChIP–qPCR analyses were performed with chromatin extracts from WT and *Jdp2* KO MEFs using specific antibodies (Ab) for Jdp2, Nrf2, MafK and IgG (as a negative control). (**b** and **d**) Schematic representations indicate the positions of primer motifs in *HO-1* (**b**) and *NQO1* promoter regions (**d**). Extracts from TPA-treated (24 h) or -untreated MEFs (5 × 10^8^ cells) from WT and *Jdp2* KO mice were subjected to ChIP assays with the indicated antibodies. Precipitated DNA and input DNA (1/20-fold) were analyzed using PCR with primers specific for E1 and E2 of the *HO-1* (**c**) and ARE of the *NQO1* (**e**) promoters. The nonspecific sequence motifs (NS) were used as a negative control (Supplementary Table SII). The data represent the mean±S.D. (*n*=3) with *P*-values from Student's unpaired *t-*tests: *P<0.05; **P<0.01

The sharing between the Nrf2-responsive gene family and the JDP2 target gene family was examined in WT MEFs and *Jdp2* KO MEFs by quantitative reverse transcription-PCR (qRT-PCR) (Figure 6a). In agreement with the protein expression data shown in Figure 2a, mRNA levels of HO-1^31^ were significantly lower in *Jdp2* KO MEFs compared with WT MEFs, as were transcript levels of the glutamate–cysteine ligase catalytic subunit (*Gclc*).^32^ Other genes such as Gclc modifier subunit (*Gclm*),^33^ jagged 1 protein (*Jag1*)^34^ and *NQO1*^35^ were reduced moderately in *Jdp2* KO MEFs. Expression of thioredoxin reductase 1, cytoplasmic (*Txnrd1*)^36^ did not differ significantly (*P*=0.07; Figure 6a).

Chromatin immunoprecipitation (ChIP)–qPCR experiments were performed to examine whether Nrf2, MafK and JDP2 are recruited *in vivo* to the ARE of the *HO-1* and *NQO1* genes in WT and *Jdp2* KO MEFs, respectively (Figures 6b and d). We designed specific primers capable of amplifying the AREs at either the enhancer E1 or E2 site of the *HO-1* transcriptional control region, each of which contains multiple stress-responsive elements^8^ and an Maf-recognition element.^37^

We first analyzed the E2 site. In WT MEFs, in the absence of TPA induction, basal levels of JDP2, MafK and Nrf2 were bound to the E2 site, and binding of all three proteins increased 2.2- to 2.5-fold in response to a 24-h TPA treatment (Figure 6c). By contrast, in *Jdp2* KO MEFs, MafK and Nrf2 were recruited to the E2 site and their bindings were stimulated 1.8- to 2.2-fold after TPA treatment. These results confirm the presence of Jdp2 at the E2 site in native chromatin and suggest that Jdp2 contributes to Nrf2 recruitment. We next analyzed the E1 site. This enhancer primarily recruits only MafK and low level of Jdp2 and Nrf2 in WT MEFs. TPA stimulates MafK binding in both WT and *Jdp2* KO MEFs. Thus, the E1 site does not seem to be dependent much on Nrf2 and Jdp2 recruitment. As expected, ChIP assays carried out with control IgG did not yield detectable recruitment of any of the above protein (Figure 6c, right side).

The third site analyzed was the ARE of the *NQO1* promoter. Similar to the E2 site of the *HO-1* gene, MafK, Jdp2 and Nrf2 were all recruited at basal levels in the absence of TPA in WT MEFs. TPA treatment increased recruitment of all three transcriptional factors by about 1.8- to 2.8-fold in WT MEFs, whereas in *Jdp2* KO MEFs both MafK and Nrf2 recruited in the uninduced state and their binding increased upon TPA treatment (1.5- to 1.7-fold; Figure 6e). As was the case for the *HO-1* gene E2 site, these results suggest that the presence of Jdp2 is critical for efficient Nrf2 recruitment to the ARE of the *NQO1* promoter.

## Discussion

In this study, we provide three lines of evidence connecting JDP2 to the transcriptional regulation of ROS-dependent gene expression. First, increased levels of ROS and 8-oxo-dGuo were detected in *Jdp2* KO MEFs and the ration of GSH/GSSG was lower compared with WT MEFs, resulting in the promotion of a more oxidized environment (Figure 1). These results indicate that JDP2 function is required to protect against intracellular oxidation, ROS activation and DNA oxidation. Second, we found that the protein and mRNA expression of the *HO-1* gene and the expression of several transcripts involved in ROS metabolism are dependent on Jdp2 (Figures 2, 3 and 6). Third, the results of the EMSA assay carried out with extracts from WT MEF cells showed that Jdp2 is present in a DNA-binding complex as one that contains MafK and Nrf2 (Figure 5d). Moreover, the results of ChIP assay also strongly argue that Jdp2 exists *in vivo* at different ARE sequences and its recruitment can be enhanced by TPA (Figure 6). Taken together, these three lines of evidence suggest that Jdp2 is an important, hitherto unidentified, component of the small Maf proteins–Nrf2 network regulating ROS-dependent gene expression.

Mechanistically, Jdp2 appears to be present at the three antioxidant AREs examined in this study under both basal and induced conditions as do MafK and Nrf2. The core sequence of the AREs from the *NQO1* and *HO-1* genes is TGACNNNGC, which matches fully the sequence of the JDP2-binding element.^14^ The results of our *in-vitro* binding studies indicate that JDP2 is able to bind DNA either as a homodimer or as a heterodimer with Nrf2 or MafK. Interestingly, JDP2 uses its basic DNA-binding domain to heterodimerize with Nrf2, while it uses its ZIP region to bind MafK. This arrangement opens up the possibility of a tripartite complex composed of JDP2, MafK and Nrf2 forming on the ARE. In support of such a tripartite complex are the results of our ChIP–qPCR analyses, showing that MafK, Nrf2 and Jdp2 were recruited together to the E2 site of the *HO-1* promoter and TPA resulted in a concerted, increased recruitment of Jdp2, MafK and Nrf2 to either the *HO-1* or *NQO1* promoter (Figures 6c and e).

Our analysis of genes whose expression is affected by the loss of JDP2 revealed that a subset of previously identified Nrf2 target genes such as *HO-1* and *NQO1*^11, 37, 38^ is also regulated by JDP2 (Figure 6a). For some of these genes, the difference in expression levels between WT and *Jdp2* KO MEFs was small, but reproducible. The clear upregulation of MafK protein levels observed in *Jdp2* KO MEFs (Figures 2a and 3b) may, in part, compensate for or mask the loss of Jdp2 in stimulating expression of these genes. In addition, the presence of a feedback loop between Jdp2 and MafK suggests that these two proteins are indeed biologically interconnected. The exact mechanism underlying this compensatory upregulation of MafK needs to be explained. It may occur at the level of transcription of the *MafK* gene or protein stabilization of MafK.^39^

Analysis of *Nrf2*-null mutant mice showed that Nrf2 is a central regulator of the induction of many antioxidant-responsive genes and enzymes, and that Nrf2 is a key transcriptional activator of the ARE.^6^ Under normal conditions, Nrf2 is captured in the cytoplasm by Keap1 and is turned over rapidly via proteasomal degradation.^6, 40, 41, 42, 43^ In response to oxidative stress, Nrf2 is stabilized, relocates to the nucleus, forms heterodimers with a small Maf protein and binds to and activates target antioxidant-response genes and genes encoding detoxification enzymes.^12, 44, 45, 46^ However, additional regulatory mechanisms for controlling nuclear Nrf2 activity must exist, as some cells contain highly constitutive nuclear levels of Nrf2, such as HeLa cells shown in Supplementary Figure S6, and MEF cells exhibit promoter-bound Nrf2 in the absence of an inducer (Figures 6c and e). Nrf2 is not a DNA-binding protein by itself, but a strong DNA-binding activity becomes evident after the association of Nrf2 with JDP2 and/or JDP2–MafK (Supplementary Figure S4). Therefore, the formation and activity of Nrf2/Jdp2/MafK DNA-binding complexes may be the targets of additional regulatory mechanisms modulating expression of ARE-dependent genes. In the absence of JDP2, MafK and Nrf2 are recruited to the ARE and, by themselves, are not able to efficiently stimulate the ARE promoter activity (Supplementary Figure S2b). Apart from JDP2 protein, JunD might have a similar function because it is associated with Nrf2 and is bound to the ARE to enhance the NRH:quinone oxidoreductase 2(*NQO2*)-reporter activity.^47^

In summary, our study demonstrates that JDP2 binds *in vivo* to the ARE and is required to increase the ARE-dependent gene expression in cooperation with Nrf2–MafK factors. In this function, JDP2 has a critical role in protecting cells or tissues against the malicious attack by exogenous carcinogens and endogenous ROS/nitrogen species by fully inducing several detoxifying or antioxidant enzymes.

## Materials and Methods

### Reagents and cell culture

Antibodies against Nrf2 (C-20x), NF-E2p18 (MafK; C-16x), *α*-tubulin (B-7), HO-1 (H-105) and NQO1 (C-19) were from Santa Cruz Biotechnology Inc. (Dallas, TX, USA). The anti-JDP2 (ab40916, lot 469784) antibody to detect the endogenous JDP2 protein was from Abcam Inc. (Cambridge, UK; see Supplementary Figures S7a and b). The anti-FLAG M2 monoclonal antibody (F1804) was obtained from Sigma-Aldrich (St. Louis, MO, USA). The polyclonal and monoclonal antibodies against JDP2 have been described elsewhere.^16, 18, 22^ Acetone, CORM, protease inhibitor mixture, TPA, tBHQ and other reagents were obtained from Sigma-Aldrich. SFN was purchased from LKT Laboratory (St. Paul, MN, USA). Human HepG2 cells were obtained from the RIKEN BRC Cell Bank (Tsukuba, Japan). The preparation of WT and *Jdp2* KO MEFs,^16, 22^ and the colony-formation assay^16^ were performed as described elsewhere.

### Plasmids

Plasmids for mouse *Jdp2*, the FLAG–*Jdp2* and its GST fusion deletion mutants were constructed as described previously.^16, 22, 24^ Nrf2 and its GST fusion deletion mutants such as NT203 (amino acids (aa) 1–203), CT313 (aa 203–516) and CT394 (aa 203–597) were amplified by PCR using *Bam*HI and *Xho*I linkers. After restriction enzyme digestion, the PCR fragment was ligated into pGEX-4T3 (Amersham Pharmacia Biotech., Little Chalfont, UK). Rat MafK and its GST fusion deletion mutants NT78 (aa 1–78) and CT78 (aa 78–156) were amplified by PCR and cloned into the *Bam*HI/*Eco*RI sites of pGEX-4T3. The FLAG–*Nrf2* and the FLAG–*MafK* were amplified by PCR and cloned into pCMV_S-FLAG vector using the respective restriction sites (RIKEN BRC DNA Bank). All recombinants were confirmed by DNA sequencing.

### Analysis of 8-oxo-dGuo, glutathione and cellular ROS

The concentrations of 8-oxo-dGuo and glutathione were measured using liquid chromatography–mass spectrometry, as described elsewhere.^48, 49^ Total GSH and GSSG concentration were calculated from a standard curve using GSSG (Cayman Chemical Co., Ann Arbor, MI, USA; 703014) prepared according to the GSH assay kit (Cayman Chemical Co.; 703002) and normalized *versus* protein concentration. Total GSH and GSSG were expressed as nmol of GSH (GSSG) of mg of protein. To measure the net intracellular accumulation of ROS, a fluorescent probe species (2′, 7′-dichlorofluorescein (DCF), the oxidation product of DCF (2′,7′-dichlorodihydrofluorescein; H_2_DCF) or that of rhodamine, the oxidation product of dihydrorhodamine 123 (DHR 123); Molecular Probes, Eugene, OR, USA) was used. After 2 h of treatment with antioxidants, cells were washed twice with HBSS solution (Gibco, Carlsbad, CA, USA) and loaded with 10 mmol/l of H_2_DCF diacetate (H_2_DCFDA) or DHR 123 in a 5% CO_2_ incubator kept at 37 °C. After 5-min incubation, cells were washed twice with HBSS (Gibco), suspended in complete medium and examined under a microscope. The number of DCF-stained cells was calculated in an area of 8.75 mm^2^.^26, 49, 50^

### Transfection and luciferase reporter assay

The double-stranded oligonucleotides corresponding to nucleotides (nt) −471 to −447, the ARE of the human *NQO1* gene were synthesized with a *Bgl*II/*Kpn*I linker, annealed, phosphorylated by T4 polynucleotide kinase and generated the reporter plasmid pGL4–hQR25–luciferase. WT and *Jdp2* KO MEFs (1 × 10^5^ cells) were plated into each well of 12-well plates and cultured for 24 h. The cells were then cotransfected with 100 ng of an ARE plasmid encoding firefly luciferase and 50 ng of the pGL4–TK plasmid encoding *Renilla* luciferase (Promega Co., Madison, WI, USA) using the Effectene Transfection Reagent kit (Qiagen, Hilden, Germany) or Lipofectamine 2000 (Invitrogen, Grand Island, NY, USA) as described in the manufacturer's protocols. For overexpression, cells were cotransfected with pcDNA3 encoding Nrf2, MafK or Jdp2. The total amount of transfected DNA was kept constant at 1 *μ*g/well by the addition of a pcDNA3 control vector. After 24 h of incubation, the cells were incubated in the presence or absence of TPA (10^−6^ M), SFN (5 × 10^−6^ M) in 0.1% dimethyl sulfoxide (DMSO) or 0.1% DMSO alone as a control for 24 h. The activities of luciferase and *Renilla* were measured in a luminometer (Berthold Technologies GmbH and Co. KG, Bad Wildbad, Germany) using the Dual-Luciferase Reporter Assay System (Promega Co.) as described elsewhere.^5, 16^ Luciferase activity values were normalized to transfection efficiency, which was monitored by *Renilla* expression, and ARE transcription activity was expressed as fold induction relative to that of the control cells.

### Small interfering RNAs

Predesigned siRNAs against human, mouse and rat Nrf2 (S9492: sense, 5′-CGUUUGUAGAUGACAAUGAtt-3′ antisense, 5′-UCAUUGUCAUCUACAAACGgg-3′), and a control scrambled siRNA (ID; 4611) were purchased from Ambion (Austin, TX, USA). The siRNAs against JDP2 and green fluorescent protein control (TRCN 0000081973, 0000081974, 0000081976 and 0000081977) were synthesized by Nippon EGT (Toyama, Japan) according to the Gene Swatter siRNA program. The sequence of the JDP2 siRNA is available from the authors on request.

### Immunoprecipitation and western blot analysis

Cells were collected using a modified RIPA buffer and a protease inhibitor cocktail (Nacalai Tesque, Kyoto, Japan). The preparation of cell lysates, SDS-polyacrylamide gel electrophoresis (SDS-PAGE; 8 or 10% gel) and western blotting were performed as described elsewhere.^16, 22, 40^ The molecular weight of Nrf2 was recently revised,^51^ and the JDP2 antibodies to detect the endogenous JDP2 were from Aronheim and colleagues^18^ and our monoclonal antibodies, as well as Abcam Inc. (ab40916, lot 469784). In the case of sequential immunoprecipitation and western blot analysis, the transformed clones of 293T cells with FLAG_S–Jdp2, FLAG_S–Nrf2 and FLAG_S–MafK were established by selection with G418 for 2 weeks. The nuclear fractions of each transformant were prepared for the sequential immunoprecipitation and western blotting as described elsewhere.^14, 16^ Each supernatant was incubated with FLAG M2 (Sigma-Aldrich; F1804) or JDP2-specific antibody, and then incubated with protein A/G-Sepharose beads (Amersham Pharmacia Biotech, Uppsala, Sweden). The beads were pelleted, washed and applied to western blotting.

### Protein–protein interaction assay

A rabbit reticulocyte lysate system (Promega Co.) was used to prepare the recombinant proteins of Nrf2, MafK and JDP2, according to the manufacturer's protocol. GST and GST fusion proteins of GST–Nrf2, GST–MafK and their deletion mutants were prepared as described elsewhere.^5, 40^ For protein–protein interaction assays, 10 *μ*l of glutathione-Sepharose beads containing 10 *μ*g of GST fusion proteins were incubated with 5 *μ*l of nonradioactive *in vitro*-translated proteins in a final volume of 500 *μ*l of binding buffer. After incubation for 2 h at 4 °C, the bead-bound protein complexes were washed extensively (five times) with wash buffer (100 mM NaCl, 20 mM 4-(2-hydroxyethyl)-1-piperazineethanesulfonic acid (HEPES), pH 7.9, 0.1% NP-40, 5 mM MgCl_2_, and 0.5 mM PMSF) followed by elution of protein complexes with SDS sample buffer and loading onto 8 or 15% SDS-PAGE. The proteins bound to GST fusion proteins were visualized by western blotting using antibodies against Nrf2, MafK or JDP2.

### Electrophoresis mobility shift assay

Five micrograms of GST fusion protein or 10 ng of *in vitro*-translated proteins was incubated at 25 °C for 30 min with T4 kinase-labeled human *NQO1*–ARE oligonucleotide (5′-CAGTCACAGTGACTCAGCAGAATCT-3′) in the presence or absence of unlabeled double-stranded mutant AREs (Supplementary Table SI). The products were resolved at 4 °C on a 5% nondenaturing polyacrylamide gel in 0.5 × Tris-borate/EDTA buffer, exposed to a radioactive imaging plate, and detected on an FLA-2000 machine (Fuji Photo Film, Tokyo, Japan) as described elsewhere.^26^ *In vivo* EMSA assay was performed as described elsewhere,^19^ with a slight modification. Nuclear extracts were prepared from WT and *Jdp2* KO MEFs that had been incubated in Dulbecco's modified Eagle's medium (DMEM, serum-free or plus 10% fetal calf serum) for 24 h. Supershift assays were performed by additional incubation with appropriate antibodies for 20 min before electrophoresis. The antibodies used were to JDP2 (174 or 249 monoclonal antibody),^16^ Nrf2 (Santa Cruz Biotechnology Inc.; C-20x), NF-E2p18 (Santa Cruz Biotechnology Inc.; MafK; C-16x) and pre-immuned rabbit IgG.^16^

### Chromatin immunoprecipitation–qPCR

ChIP assays were performed as described by Kotake *et al.*,^52^ with modification of the washing conditions. The immunoprecipitated protein–DNA complexes were washed twice with binding buffer (10 mM HEPES, pH 7.9, 10 mM Tris-HCl, pH 7.9, 12.5% glycerol, 0.25% NP-40, 0.5% Triton X-100, 0.24 M NaCl, 0.75 mM MgCl2, 1.1 mM EDTA and protease inhibitor mixture) and then washed twice with Tris-EDTA buffer (10 mM Tris-HCl, pH 7.9, 1 mM EDTA). The protein–DNA complexes were disrupted with proteinase K (Sigma-Aldrich) at pH 6.8. DNA was extracted with phenol and chloroform, precipitated in ethanol and analyzed by real-time PCR using the Power SYBR Green PCR Master Mix (Life Technologies, Carlsbad, CA, USA) and the primers shown in Supplementary Table SII. A qPCR was performed as described below.

### Isolation of RNA, RT-PCR and real-time qRT-PCR analysis

Total RNA was extracted from various tissues of both *Jdp2* KO and WT adult mice, from embryos and from corresponding MEFs treated with TRIzol (Invitrogen) according to the manufacturer's instructions. Expression of JDP2 mRNA was analyzed by northern blotting as described elsewhere.^15^ qRT-PCR was performed on a PRISM 7700 system (Amersham Biosystems, Foster City, CA, USA) according to the manufacturer's instructions. We designed the primers using the public-domain Primer 3 program of GENETYX-Mac Ver.14 software (Hitachi Software, Tokyo, Japan). The respective pairs of primers are listed in Supplementary Table Sll. In the case of real-time qPCR, total RNA samples (0.5–1.0 *μ*g) were reverse-transcribed with GenAmp Gold RNA PCR Core kit (4308207, Applied Biosystems, Grand Island, NY, USA) and the resulting cDNA samples (1 *μ*l) were amplified with the specific primer pairs (see Supplementary Table SIII) using the following temperature cycles: 10 min initial denaturation at 95 °C; 40 cycles, 15 s denaturation at 95 °C and 1 min annealing at 60 °C. The mRNA levels were determined by real-time PCR using Power SYBER Green PCR Master Mix (Life Technologies) and the relative gene expression was calculated using GAPDH mRNA as control housekeeping.

### Immunofluorescence

Cells were cultured in Iscove's modified DMEM containing 1 and 4% bovine serum albumin (BSA) at 37 ^o^C. After *in-situ* extraction with 0.1% Triton X-100 at 4 ^°^C for 3 min, cells were fixed in 4% paraformaldehyde for 20 min and permeabilized in phosphate-buffered saline (PBS) containing 0.1% saponin and 3% BSA at room temperature.^16^ Cells were stained with anti-JDP2 (monoclonal antibodies 249 or 176)^16, 22^ and anti-Nrf2 (Santa Cruz Biotechnology Inc.) or anti-MafK (Santa Cruz Biotechnology Inc.) antibodies for 2 h, washed with PBS containing 0.1% saponin and stained with Alexa Fluor 488-conjugated secondary antibody or Alexa Fluor 546-conjugated secondary antibody (Life Technologies) for 1 h. For DNA staining, cells were treated with 200 *μ*g/ml RNase A for 30 min and 25 ng/ml TOPRO-3 (Life Technologies) for 30 min. Stained cells were mounted with antifade reagent. Confocal and Nomarski differential-interference-contrast images were obtained using an FV500 laser-scanning microscope (Olympus, Tokyo, Japan) as described previously.^53^

### TPA-induced epidermal thickening *in vivo*

Epidermal thickening was measured as described previously.^16^ Briefly, the dorsal skin of each mouse (six pairs of WT and KO mice) was painted with TPA twice at an interval of 24 h (8.1 nmol in 100 *μ*l acetone; Sigma-Aldrich) or with AFN (15 nmol in 100 *μ*l acetone) applied topically. Mouse skin was collected 1 h after the second TPA or SFN treatment. The tissues were fixed with 4% paraformaldehyde, embedded in paraffin and sectioned at a thickness of 5 *μ*m. The epidermal thickness of the skin was measured at 10 sites of a randomly selected region using an Olympus microscope (Olympus). The vertical thickness of the epidermis was defined as the distance from the stratum basal to the stratum corneum. All mice were housed at the National Laboratory Animal Center mouse facilities in accordance with the Institutional Animal Care and Use Committee.

### Statistical analyses

Differences between the treatments and the control were identified using one-way analysis of variance and SPSS-16 Software (IBM Co., Armonk, NY, USA). The data are presented with S.E.M. of three to five samples per assay. Comparisons were made using a two-tailed Student's *t*-test for the difference in mean between two groups. A *P*-value probability of <0.05 was considered significant.

## Abbreviations

ARE: antioxidant-responsive element
bZIP: basic zipper region
ChIP: chromatin immunoprecipitation
HO-1: hemoxigenase-1
GST: glutathione *S*-transferase
JDP2: Jun dimerization protein 2
KO: knockout
MEFs: mouse embryonic fibroblasts
Nrf2: nuclear factor-erythroid 2-related factor 2
NQO1: NAD(P)H dehydrogenase quinone 1
qPCR: quantitative PCR
ROS: reactive oxygen species
siRNA: small interfering RNA
TPA: 12-*O*-tetradecanoylphorbol-13-acetate
