# Supplementary material for: Jun dimerization protein 2 is a critical component of the Nrf2/MafK complex regulating the response to ROS homeostasis
Source: Cell Death Dis. 2013 Nov 14;4(11):e921–. doi: 10.1038/cddis.2013.448 (PMC3847324; doi:10.1038/cddis.2013.448)
Supplement: Supplementary Information [file cddis2013448x1.pdf]

Supplementary information

Supplementary methods

Reagents and cell culture. Antibodies against Nrf2 (C-20x), NF-E2p18 (MafK; C-16x), were from Santa Cruz Biotechnology Inc. (Santa Cruz Biotech Inc., Dallas, TX). The anti-JDP2 (ab40916, lot # 469784) antibody to detect the endogenous JDP2 protein was from Abcam (Cambridge, UK: see Supplementary Figure S7a and b). The heteroantibodies against JDP2 and monoclonal antibodies have been described elsewhere.[1-3] Protease inhibitor mixtures, 12-*O*-tetradecanoylphorbol-13-acetate (TPA), sulforaphane (SFN), and tertiary butyl hydroquinone (tBHQ) were obtained from Sigma-Aldrich (St. Louis, MO, USA). Human HepG2 cells were obtained from the RIKEN BRC Cell Bank (Tsukuba, Japan). The preparation of WT and *Jdp2* KO MEFs,[1,3] and the colony-formation assay[1] were performed as described.

Plasmid molecules. FLAG–*Nrf2*, FLAG–*MafK* and FLAG-*Jdp2* were amplified by PCR and cloned into pCMV_S-FLAG vector using the respective restriction sites (RIKEN BRC DNA Bank, Tsukuba, Japan). All recombinants were confirmed by DNA sequencing.

Analysis of glutathione and ROS production. The concentrations of glutathione were measured using liquid chromatography–mass spectrometry, as described elsewhere.[4,5] Total GSH and GSSG concentration were calculated from a standard curve using GSSG (Cayman; 703014) prepared according to the GSH assay kit (Cayman Chemical Co., Ann Arbor, MI, USA; 703002) and normalized versus protein concentration. Total GSH and GSSG were expressed as nmol of GSH (GSSG) of mg of

protein.

Transient transfection and luciferase reporter assay. The activity of *Renilla* luciferase was measured as described elsewhere.[1,6,7] WT and *Jdp2* KO MEFs (1×10^5 cells) were plated into each well of 12-well plates, and the plates were cultured for 24 h. The cells were cotransfected with the indicated amount of pGL4–hQR25–firefly luciferase reporter and pGL4–TK plasmid encoding *Renilla* luciferase (Promega, Madison, WI) in the presence or absence of pcDNA3 or pCMV_S-FLAG encoding Nrf2, MafK, or Jdp2 using the Effectene Transfection Reagent kit (Qiagen Inc., Gaithersburg, MD, USA) or Lipofectamin 2000 (Invitrogen Co., Grand Island, NY, USA). After 24 h of incubation, the cells were incubated in the presence or absence of 10^{-6} M TPA , SFN (5×10^{-6}M), or tBHQ (5×10^{-6}M) in 0.1% dimethyl sulfoxide (DMSO) or 0.1% DMSO alone as a control for 24 h.

Immunoprecipitation and Western blot analysis. Cells were harvested using a modified RIPA buffer and a protease inhibitor cocktail (Nacalai Tesque, Kyoto, Japan). The preparation of cell lysates, sodium dodecyl sulfate-polyacrylamide gel electrophoresis (SDS-PAGE) (8% or 10% gel) and Western blotting were performed as described elsewhere.[1,6,7] The molecular weight of Nrf2 was recently revised,[9] and the JDP2 antibodies to detect the endogenous JDP2 were from Aronheim's[10] and our monoclonal antibodies, as well as Abcam Inc. (ab40916, lot # 469784). In the case of sequential immunoprecipitation and Western blot analysis, the transformed clones of 293T cells with FLAG_S–*Jdp2*, FLAG_S–*Nrf2* and FLAG_S–*MafK* were established by selection with G418 for two weeks. The nuclear fractions of each transformant were

prepared for the sequential immunoprecipitation and Western blotting as described elsewhere.[1,11] Each supernatant was incubated with FLAG M2 (Sigma-Aldrich; F1804) or JDP2-specific antibody, and then incubated with protein A/G-Sepharose beads (Amersham Pharmacia Biotech, Uppsala, Sweden). The beads were pelleted, washed, and applied to Western blotting.

Protein–protein interaction assay. A rabbit reticulocyte lysate system (Promega, Madison, WI) was used to prepare the recombinant proteins of Nrf2, MafK, and JDP2, according to the manufacturer's protocol. GST and GST-fusion proteins of GST–Nrf2, GST–MafK, and their deletion mutants were prepared as described elsewhere.[6,7] For protein–protein interaction assays, 10 μL of glutathione-Sepharose beads containing 10 μg of GST–fusion proteins were incubated with 5 μL of nonradioactive *in vitro*-translated proteins in a final volume of 500 μL of binding buffer. After incubation for 2 h at 4°C, the bead-bound protein complexes were washed extensively (five times) with wash buffer [100 mM NaCl, 20 mM 4-(2-hydroxyethyl)-1-piperazineethanesulfonic acid (HEPES), pH 7.9, 0.1% NP-40, 5 mM $MgCl_2$, and 0.5 mM PMSF], followed by elution of protein complexes with SDS sample buffer and loading onto 8% or 15% SDS-PAGE. The proteins bound to GST-fusion proteins were visualized by Western blotting using antibodies against Nrf2, MafK, or JDP2.

EMSA. Five micrograms of GST fusion protein or 10 ng of *in vitro*-translated proteins was incubated at 25°C for 30 min with T4 kinase-labeled human NQO1-ARE oligonucleotide (5′-CAGTCACAGTGACTCAGCAGAATCT-3′) in the presence or

absence of unlabeled double-stranded mutant AREs (Supplementary Table SI). The products were resolved at 4°C on a 5% nondenaturing polyacrylamide gel in 0.5 × Tris-borate/EDTA buffer, exposed to a radioactive imaging plate, and detected on an FLA-2000 machine (Fuji Photo Film, Tokyo, Japan) as described elsewhere.[6,7] *In vivo* EMSA was performed as described elsewhere,[6,7] with slight modification. Nuclear extracts were prepared from WT and *Jdp2* KO MEFs that had been incubated in Dulbecco's modified Eagle's medium (DMEM, serum-free or plus 10% fetal calf serum) for 24 h. Supershift assays were performed by additional incubation with appropriate antibodies for 20 min before electrophoresis. The antibodies used were to JDP2 (174 or 249 monoclonal antibody; Abcom; ab40916, #469784),[1,8] Nrf2 (Santa Cruz; C-20x), NF-E2p18 (Santa Cruz; MafK; C-16x), and preimmune rabbit IgG.[6,7]

ChIP. ChIP assays were performed as described by Kotake *et al.*,[12] with modification of the washing conditions. The immunoprecipitated protein–DNA complexes were washed twice with binding buffer (10 mM HEPES, pH 7.9, 10 mM Tris-HCl, pH 7.9, 12.5% glycerol, 0.25% NP-40, 0.5% Triton X-100, 0.24 M NaCl, 0.75 mM MgCl2, 1.1 mM EDTA, and protease inhibitor mixture) and then washed twice with Tris-EDTA buffer (10 mM Tris-HCl, pH 7.9, 1 mM EDTA). The protein–DNA complexes were disrupted with proteinase K (Sigma-Aldrich) at pH 6.8. DNA was extracted with phenol and chloroform, precipitated in ethanol, and analyzed by real-time PCR using the Power SYBR® Green Master Mix and the primers shown in Supplementary Table SII.

Real-time PCR. Total RNA was extracted from WT MEFs and *Jdp2* KO MEFs as described above. Total RNA samples (0.5–1.0 μg) were reversed–transcribed with

GenAmp Gold RNA PCR Core kit (4308207, Applied Biosystems, Grand Island, NY) and the resulting cDNA samples (1 μL) were amplified with the specific primer pairs (see Supplemental Table SIII) using the following temperature cycles: 10 min initial denaturation at 95°C; 40 cycles, 15-s denaturation at 95°C and 1 min annealing at 60°C. The mRNA levels were determined by real-time PCR using Power SYBER green (4367659; applied Niosystem9 in an Applied Biosystems detector, and the relative gene expression was calculated using GAPDH mRNA as control housekeeping.

Statistical analyses. Differences between the treatments and the control were identified using one-way analysis of variance and SPSS-16 Software (IBM Co., Armonl, NY). The data are presented with standard error of the mean (SEM) of 3–5 samples per assay. Comparisons were made using a two-tailed Student's *t*-test for repeated measures. A p value probability of < 0.05 was considered significant.

Supplementary Figures

Figure S1. Differential responses of antioxidation reactions in WT MEFs and Jdp2 KO MEFs. (a) Determination of the GSH/GSSG ratio in WT and *Jdp2* KO MEFs incubated with TPA (10^{-6} M) for 24 h. The ration of GSH/GSSG was calculated in the Materials and Methods. Data are presented as mean ± SD (n = 3). The data were analyzed using Student's *t* test. ** $p < 0.01$.

Figure S2. ARE-driven transcriptional activity in WT and *Jdp2* KO MEFs. (a) Effect of CORM on NQO1 promoter activity. WT and *Jdp2* KO MEFs were exposed to TPA (10^{-6} M) alone or in the presence of 50 nmol/L of CORM. After 24 h of culture, luciferase

activity was measured. Each value represents the mean ± SD (n = 3). * $p < 0.05$; ** $p < 0.01$. (b) Effect of MafK on ARE activity in the presence of Nrf2. WT and *Jdp2* KO MEFs (5×10^4) were transfected with 400 ng of pGL4–hQR25–luciferase, 50 ng of pcDNA3-Nrf2, and the indicated amounts of pcDNA-MafK. One day after transfection, cells were harvested and assayed for luciferase activity. Each value represents the mean ± SD (n = 3). * $p < 0.05$; ** $p < 0.01$. (c, d) Effect of ARE activity in the presence of WT and *Jdp2* KO MEFs. WT and *Jdp2* KO MEFs (5×10^4) were transfected with 400 ng of pGL4–hQR25–luciferase and the indicated amounts of pcDNA-Jdp2 (c) or pcDNA-MafK (d), respectively. One day after transfection, cells were harvested and assayed for luciferase activity. Cellular luciferase activity was measured as described in Materials and methods. Each value represents the mean ± SD (n = 3).

Figure S3. Effect of siRNA specific for Nrf2 and JDP2 on ARE activity. WT and *Jdp2* KO MEFs (5×10^4 cells) were transfected with 30 pmole of siRNA specific for Nrf2 or JDP2 and 200 ng of pGL4–hQR25–luciferase plasmid, and then exposed to 10^{-6} M TPA, as described in the text. After exposure for 30 h, luciferase activity was measured (n = 3). The same amount of nonspecific double-stranded RNA was used as a negative control (NS). **, $p < 0.01$.

Figure S4. Cooperative binding of JDP2, MafK and Nrf2 to ARE. EMSA reactions were carried out as described in Materials and Methods using [γ-^{32}P]-labeled double-stranded ARE oligonucleotides. IVT-Jdp2, IVT-MafK, and IVT-Nrf2 were expressed using an *in vitro* transcription/translation system without [^{35}S]-methionine. The reaction mixture of the ARE probe was incubated with the indicated amounts (2.5,

5.0, and 10.0 ng) of IVT-Jdp2, IVT-MafK, or IVT-Nrf2. EMSA was performed and the results were analyzed on native gels (5% polyacrylamide), as described in Materials and Methods. The amount of each IVT protein was indicated as 1 ng or 2 ng. “Bound” indicates the supershifted DNA-protein complexes, and “Free” indicates the ARE DNA probe.

Figure S4. Cooperative binding of JDP2, MafK and Nrf2 to ARE. EMSA reactions were carried out as described in the “Materials and Methods” using [γ-^{32}P]-labeled double-stranded ARE oligonucleotides. IVT-JDP2, IVT-MafK, and IVT-Nrf2 were expressed using an *in vitro* transcription/translation system without [^{35}S]-methionine. The reaction mixture of the ARE probe was incubated with the indicated varing amounts (approximately 2.5, 5.0, and 10.0 ng) of IVT-JDP2, IVT-MafK, or/and IVT-Nrf2. EMSA was performed and the results were analyzed on native gels (5% polyacrylamide), as described in the “Materials and Methods”.

Figure S5. Association complex of Nrf2, MafK and JDP2 in transformed 293T cells. pFLAG_S-Jdp2, pFLAG_S-MafK, pFLAG_S-Nrf2, and pFLAG_S-SP-1 (10 μg each) were transfected into human HEK293T cells, and selected with G418 as described elsewhere.[1] Nuclear extracts from cells (2×10^8 cells) were prepared, and then the immunoprecipitation and Western blotting was performed. Immunoprecipitates of antibody specific for FLAG M2-agarose beads of protein A/B (Invitrogen; 50 μl each) and then released with SDS-PAGE sample buffers and then the Western blotting were carried out by antibodies against Nrf2, MafK, Jdp2, and β-actin. Lane 1; co-immunoprecipitation experiments demonstrated 1% of input total extracts. The

immunoprecipitated proteins with anti-FLAG were visualized with antibody specific FLAG and control β-actin.

Figure S6. Colocalization of JDP2 with Nrf2 or MafK. HeLa cells were triply stained with anti-JDP2, anti-Nrf2 (JDP2–Nrf2; panel a), or anti-MafK (JDP2–MafK; panel b) antibodies and with TOPRO-3. The resulting red emission of TOPRO-3-stained nuclei is pseudocolored white. Scale bars, 10 μm.

Supplementary Figure S7. Detection of endogenous JDP2 protein. Endogenous Jdp2 protein was detected by immunoblotting as described in Materials and Methods. (a) Recombinant Flag-tagged Jdp2 protein was synthesized using an *in vitro* translation system and was used as the positive control (lane 1). Flag-tagged Jdp2 and Flag-vector only were expressed in HepG2 cells (lane 4 and 3). HepG2 cells without transfection (lane 2). (b) Endogenous expression of Jdp2 protein in mouse wild type embryonic fibroblasts (+/+) and Jdp2 knockout embryonic fibroblasts (-/-). NS indicated the non-specific proteins. Flag-Jdp2 and endogenous Jdp2 proteins were indicated in the right. Endogenous JDP2 protein from the total cell lysates was detected only by this heteroantibody (ab40916; lot no. 469784 only).

Supplementary Table I. Oligonucleotides of wild type and mutant of ARE.

Supplementary Table II. Sequences of primers for Nrf2-responsive genes for qPCR.

Supplementary Table III. Sequences of primers for ChIP-qPCR.

References

1. Pan J, Nakade K, Huang YC, Zhu ZW, Masuzaki S, Hasegawa, H *et al.* Suppression of cell-cycle progression by Jun dimerization protein-2 (JDP2) involves downregulation of cyclin-A2. *Oncogene* 2010; **29**: 6245–6256.
2. Weidenfeld-Baranboim K, Bitton-Worms K, Aronheim A. TRE-dependent transcription activation by JDP2-CHOP10 association. *Nucleic Acids Res* 2008; **36**: 3608–3619.
3. Katz S, Heinrich R, Aronheim A. The AP-1 repressor, JDP2, is a bona fide substrate for the c-Jun N-terminal kinase. *FEBS letters* 2001; **506**: 196–200.
4. Zhu P, Oe T, Blair IA. Determination of cellular redox status by stable isotope dilution liquid chromatography/mass spectrometry analysis of glutathione and glutathione disulfide. *Rapid Comm Mass Spec* 2008; **22**: 432–440.
5. Park JH, Mangal D, Tacka KA, Quinn AM, Harvey RG, Blair IA *et al*. Evidence for the aldo-keto reductase pathway of polycyclic aromatic *trans*-dihydrodiol activation in human lung A549 cells. *Proc Natl Acad Sci USA* 2008; **105**: 6846-6851
6. Hou DX, Fukuda M, Johnson JA, Miyamori K, Ushikai M, Fujii M. Fisetin induces transcription of NADPH:quinone oxidoreductase gene through an antioxidant responsive element-involved activation. *Int J Oncology* 2001; **18**: 1175–1179.
7. Tanigawa S, Fujii M, Hou DX. Action of Nrf2 and Keap1 in ARE-mediated NQO1 expression by quercetin. *Free Redic Biol Med* 2007; **42**:1690–1703.
8. Nakade K, Pan J, Yoshiki A, Ugai H, Kimura M, Li H *et al.* JDP2 suppresses adipocyte differentiation by regulating histone acetylation. *Cell Death Differ* 2007; **14**: 1398-1405.
9. Lau A, Tian W, Whitman SA, Zhang DD. The predicted molecular weight of Nrf2: it is what it is not. *Antioxi Redox Signal* 2013; **18**: 91-93.
10. Weidenfeld-Baranboim K, Bitton-Worms K, Aronheim A. TRE-dependent transcription activation by JDP2-CHOP10 association. *Nucleic Acids Res* 2008; **36**: 3608–3619.
11. Aronheim A, Zandi E, Hennemann H, Elledge SJ, Karin M. Isolation of an AP-1 repressor by a novel method for detecting protein-protein interactions. *Mol Cell Biol* 1997; **17**: 3094–3102.
12. Kotake Y, Cao R, Viatour P, Sage J, Zhang Y, Xiong Y. pRB family proteins are required for H3K27 trimethylation and Polycomb repression complexes binding to and silencing $p16^{INK4alpha}$ tumor suppressor gene. *Gene Dev* 2007**;** **21**: 49–54.

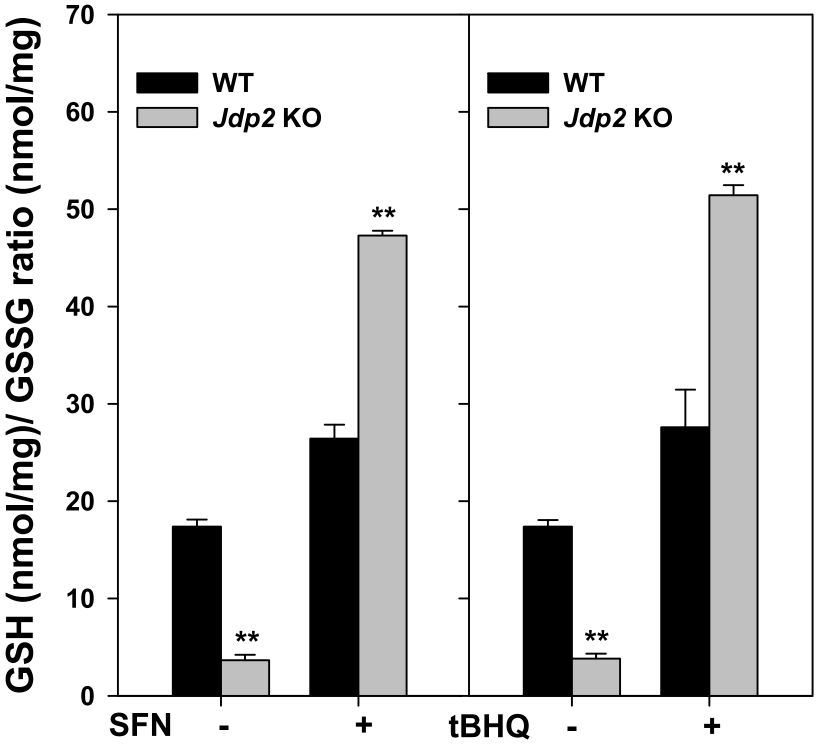

Supplementary Figure S1

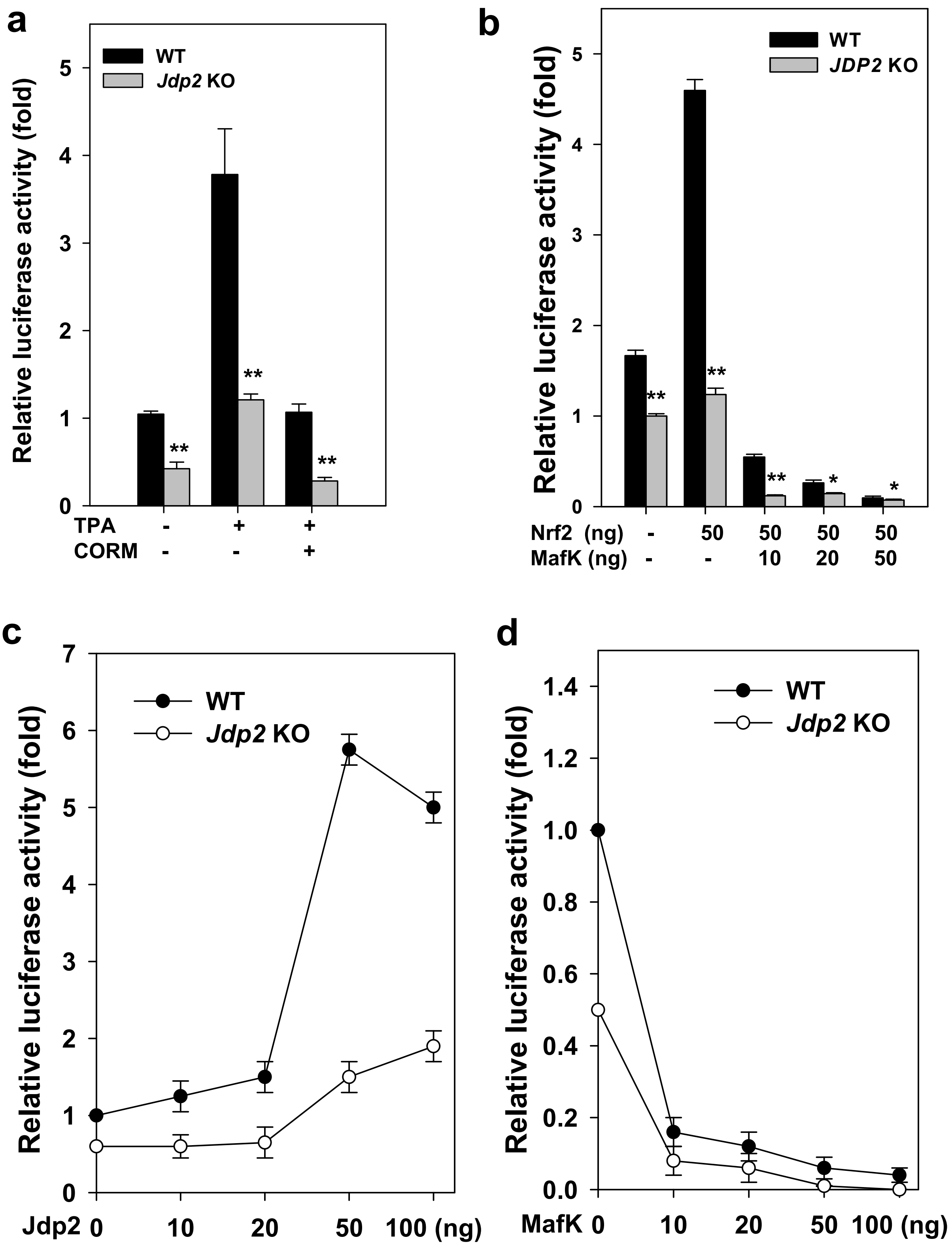

Supplementary Figure S2

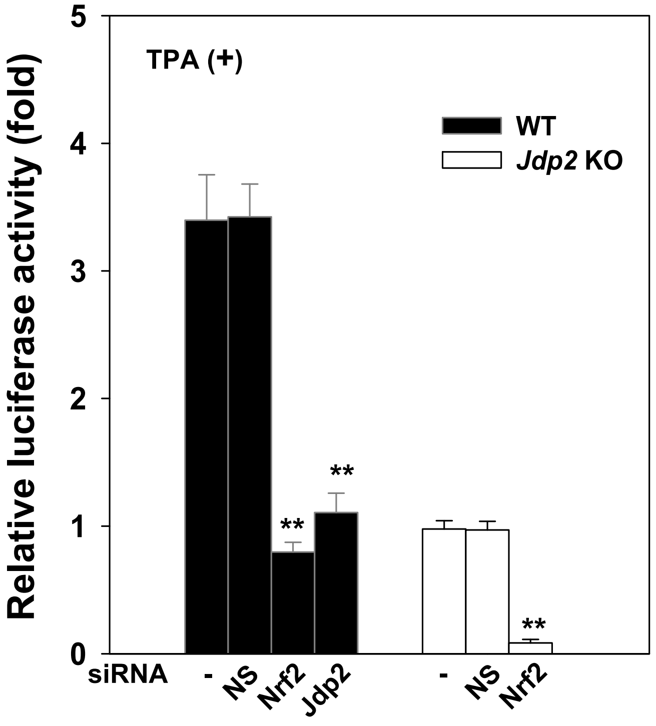

Supplementary Figure S3

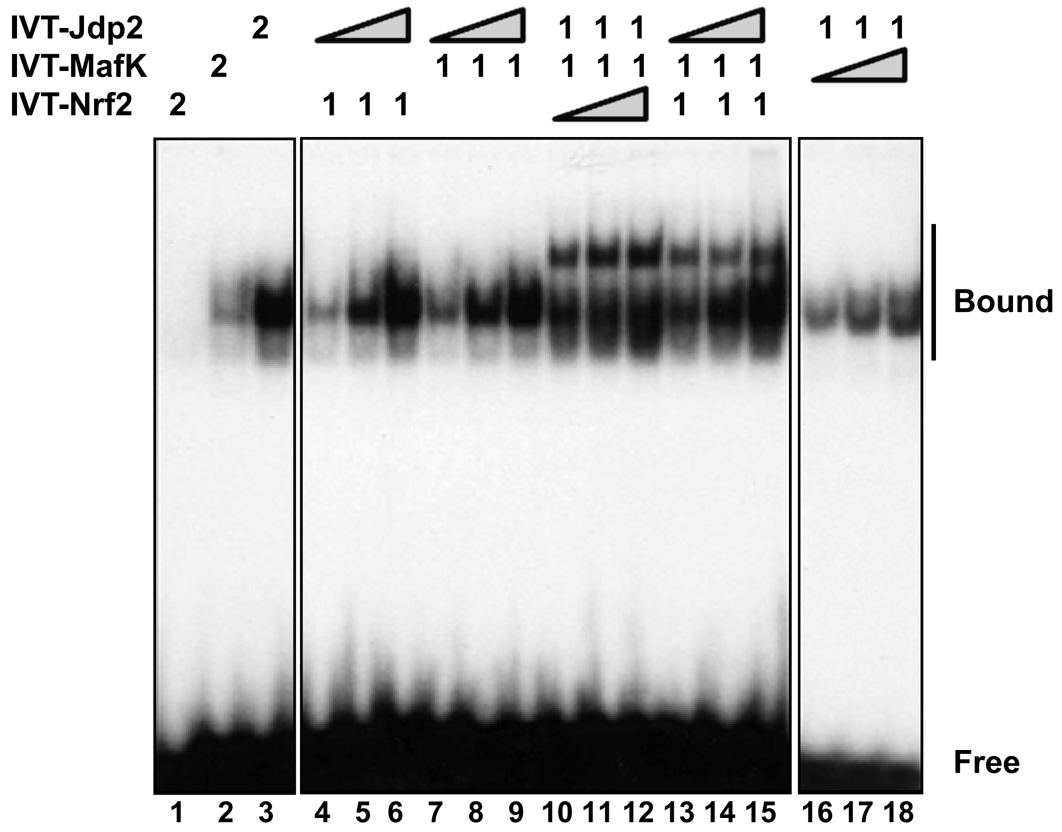

Supplementary Figure S4

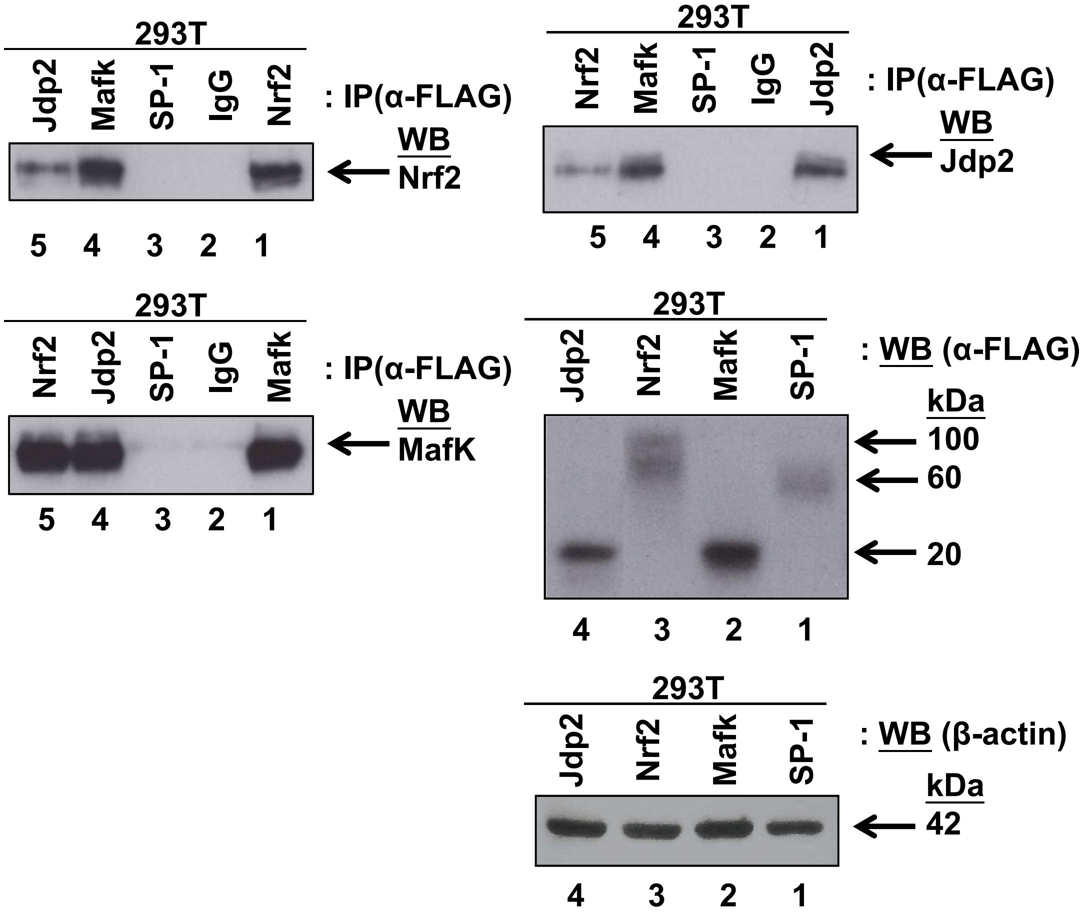

Supplementary Figure S5

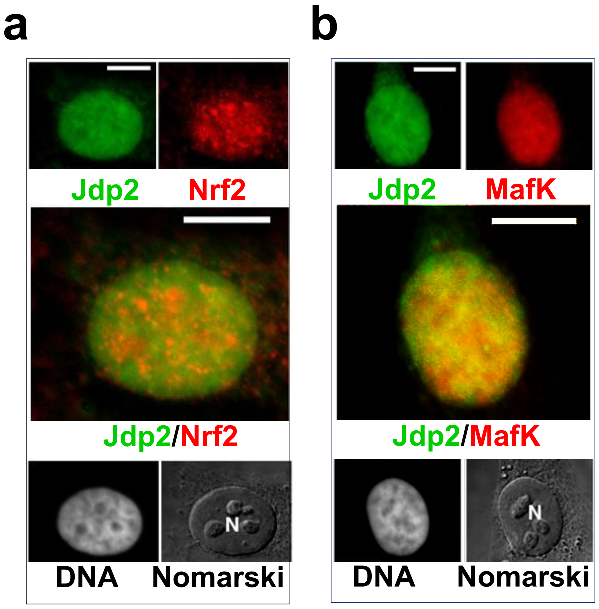

Supplementary Figure S6

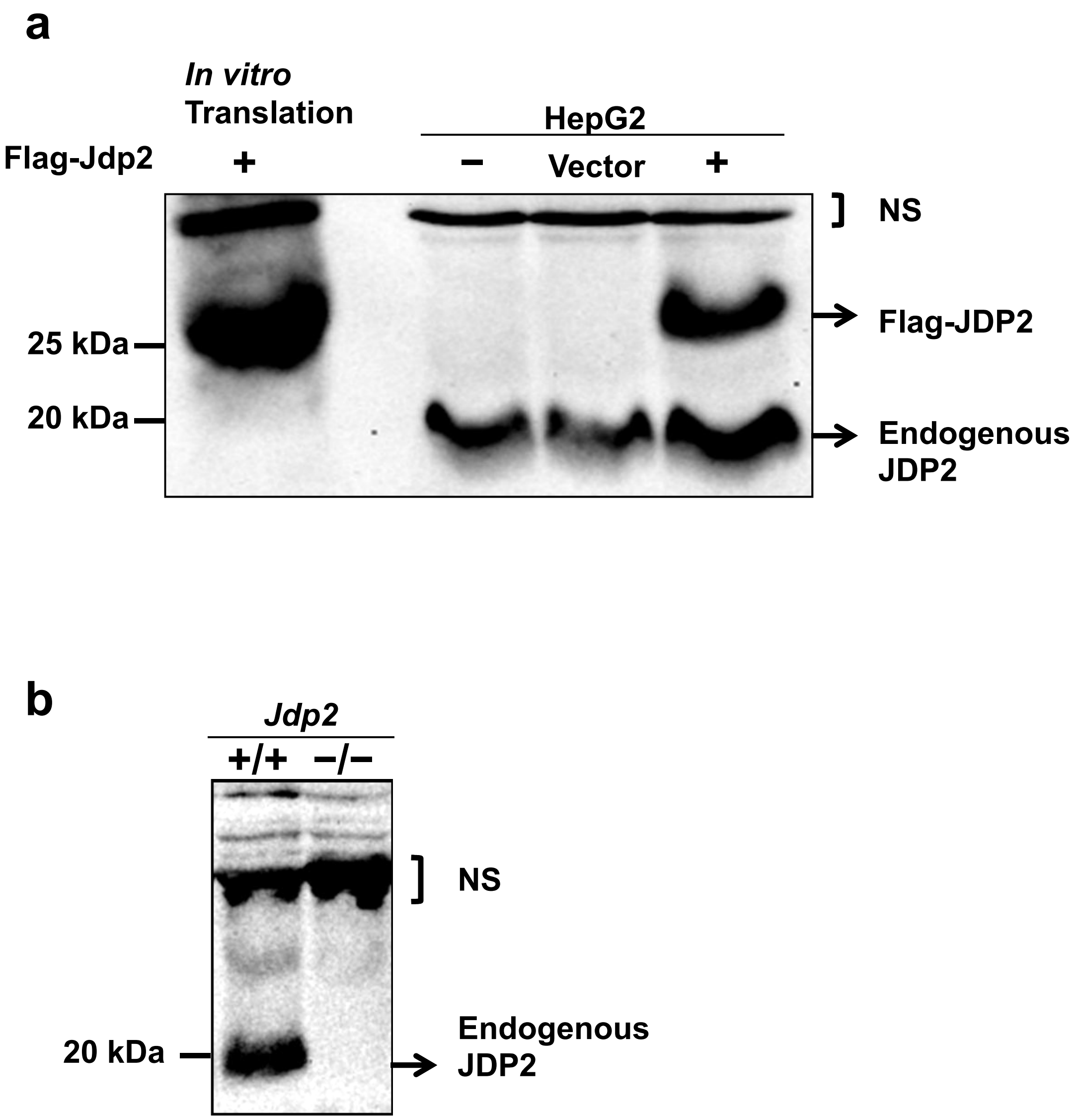

Supplementary Figure S7

Oligonucleotides of wild type (WT) and mutant (M1 to M7) of ARE

ARE		AP-1 like		ARE core	GC box	
		-471 (nt)				-447 (nt)
**WT**	5' -	C A G T C A C	A G T	G A C T C A	G C A	G A A T C T - 3'
**M1**	5' -	C A A T T A C	A A T	G A C T C A	G C A	G A A T C T - 3'
**M2**	5' -	C A G T C A C	A G A	T T C C A A	G C A	G A A T C T - 3'
**M3**	5' -	C A G T C A C	A G T	G A C T C A	T T A	G A A T C T - 3'
**M4**	5' -	C A G T C A C	A G A	T T C C A A	T T A	G A A T C T - 3'
**M5**	5' -	C A A T T A C	A A T	G A C T C A	T T A	G A A T C T - 3'
**M6**	5' -	C A A T T A C	A A A	T T C C A A	G C A	G A A T C T - 3'
**M7**	5' -	C A A T T A C	A A A	T T C C A A	T T A	G A A T C T - 3'

Mutated bases are underlined.

Supplementary Table I

Sequences of primers for chromatin immunoprecipitation-qPCR

Name	Sequence
Mouse HO-1 promoter E1	5' - T G A A G T T A A A G C C G T T C C G G - 3'
	5' - A G C G G C T G G A A T G C T G A G T - 3'
Mouse HO-1 promoter E2	5' - G G G C T A G C A T G C G A A G T G A G - 3'
	5' - A G A C T C C G C C C T A A G G G T T C - 3'
Mouse HO-1 promoter NS	5' - C T G G A G T G A G G A T T T G A T T T T T G - 3'
	5' - A A G G G A G G T C A G A G G A C A G T T T - 3'
Mouse NQO1 promoter	5' - G C A C G A A T T C A T T T C A C A C G A G G - 3'
	5' - G G A A G T C A C C T T T G C A C G C T A G - 3'
Mouse NQO1 promoter NS	5' - A G G A C C C T A T C T A G C T C C A T T - 3'
	5' - C T A C G G C A C A A C T G T A A T - 3'

Supplementary Table II

Primer sequences of mouse Nrf2–responsive genes for qPCR

Gene	mouse sequence		Ac No	size	exon junction position
NQO1	Fw	ATCACAGGTGAGCTGAAGGA	NM_008706	104 bp	exon 1/2
	Re	TTGTGTTCGGCCACAATATC			
HO-1	Fw	TCAACATTGAGCTGTTTGAGG	NM_010442	160 bp	exon 3/5
	Re	CTAGTGCTGATCTGGGGTTT			
Txnrd1	Fw	GGTGGTTTCACCTTCCTTGT	NM_001042513.1	142 bp	exon 3/5
	Re	TTTTTGTTCGGCTTCAGGGC			
GSTA4	Fw	TACTTTAATGGCAGGGGACG	NM_010357	129 bp	exon 2/3
	Re	AAGCAGGTGTCCATCCTTTT			
GSTM1	Fw	GTTACAACCCTGACTTTGAGAA	NM_010358	165 bp	exon 6/7
	Re	ACATACGGTACTGGTCAAGA			
Gclc	Fw	GACCAATGGAGGTGCAGTTA	NM_010295	142 bp	exon 11/13
	Re	GCCACTTTCATGTTCTCGTC			
Gclm	Fw	GGCACAGGTAAAACCCAATAG	NM_008129	147 bp	exon 6/7
	Re	TGGAAACTTGCCTCAGAGAG			
IGF1	Fw	CCACTGAAGCCTACAAAAGC	NM_001111274	132 bp	exon 4/6
	Re	TGTACTTCCTTTCCTTCTCCT			
JAG1	Fw	AGTCCCCATCCTTGTTACAATA	NM_013822	123 bp	exon 18/20
	Re	GGAAGACTGGCACTCATTGAT			
GAPDH	Fw	GCTCATTTCCTGGTATGACAAT	NM_008084.2	138 bp	exon 6/7
	Re	TGGGATAGGGCCTCTCTTG			

Supplementary Table III
